# Insights into the Effect of Metal Ratio on Cooperative Redox Enhancement Effects over Au- and Pd-Mediated Alcohol Oxidation

**DOI:** 10.1021/acscatal.2c06284

**Published:** 2023-02-10

**Authors:** Liang Zhao, Ouardia Akdim, Xiaoyang Huang, Kai Wang, Mark Douthwaite, Samuel Pattisson, Richard J. Lewis, Runjia Lin, Bingqing Yao, David J. Morgan, Greg Shaw, Qian He, Donald Bethell, Steven McIntosh, Christopher J. Kiely, Graham J. Hutchings

**Affiliations:** † Max Planck- Cardiff Centre on the Fundamentals of Heterogeneous Catalysis FUNCAT, Cardiff Catalysis Institute, School of Chemistry, Cardiff University, Cardiff CF10 3AT, U.K.; ‡ Department of Materials Science and Engineering, Faculty of Engineering, National University of Singapore, 119077 Singapore; § Department of Chemical and Biomolecular Engineering, Lehigh University, Bethlehem, Pennsylvania 18015, United States; ∥ Department of Materials Science and Engineering, Lehigh University, Bethlehem, Pennsylvania 18015, United States

**Keywords:** cooperative redox enhancement, oxidative dehydrogenation, oxygen reduction reaction, AuPd, biomass

## Abstract

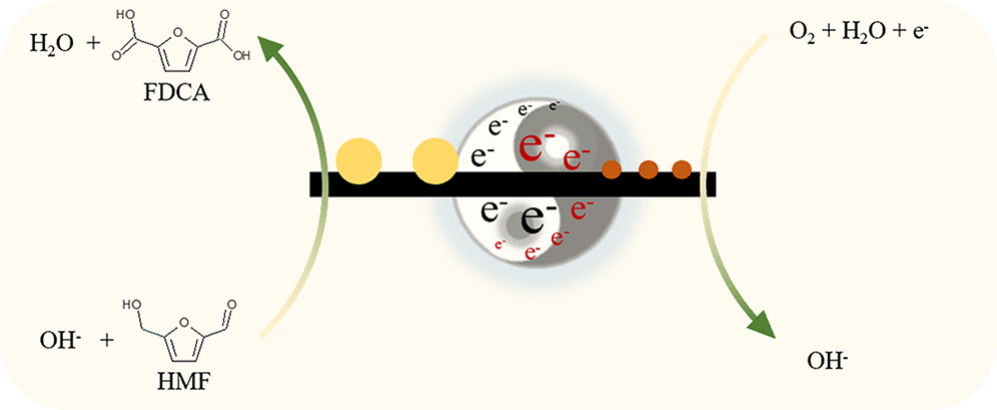

The aerobic oxidation of alcohols and aldehydes over supported heterogeneous catalysts can be considered as comprising two complementary and linked processes: dehydrogenation and oxygen reduction. Significant rate enhancements can be observed when these processes are catalyzed by independent active sites, coupled by electron transport between the two catalysts. This effect, termed cooperative redox enhancement (CORE), could significantly influence how researchers approach catalyst design, but a greater understanding of the factors which influence it is required. Herein, we demonstrate that the Au/Pd ratio used in physical mixtures of monometallic catalysts and phase-separated Au and Pd bimetallic catalysts dramatically influences the degree to which CORE effects can promote alcohol oxidation. Perhaps more interestingly, the roles of Au and Pd in this coupled system are determined to be interchangeable. Preliminarily, we hypothesize that this is attributed to the relative rates of the coupled reactions and demonstrate how physical properties can influence this. This deeper understanding of the factors which influence CORE is an important development in bimetallic catalysis.

## Introduction

The oxidation of alcohols and aldehydes are, industrially, very important chemical transformations. Supported metal catalysts have proven to be highly effective at catalyzing these reactions and are an area that has been extensively studied over the last 30 years.^1,2^ A recent review highlighted that both catalytic properties and the reaction conditions employed can dramatically influence catalyst performance in these reactions.^3^

Combining two (or more) metals, to form supported multimetallic alloy catalysts, is another approach that can be adopted to improve the rate of such reactions. Hutchings and co-workers were the first to demonstrate that AuPd bimetallic catalysts were more active than their analogous monometallic components for alcohol oxidation.^4^ Since this seminal publication, a significant amount of other research has been conducted in this field.^5−11^ Synergistic effects, which can arise from electronic or structural modifications, have been observed with many different metal combinations in a variety of different reactions.^12−14^ For the aerobic oxidation of alcohols, the ratio of the different metal components can dramatically influence the extent of the enhancement.^3,4^

Over a decade ago, Davis and co-workers demonstrated that two reactions, the oxygen reduction reaction (ORR) and dehydrogenation (DH), proceed simultaneously when an alcohol is reacted (aerobically) over a supported monometallic Au catalyst.^15^ The authors established that the two processes were complementary, confirming that the role of O_2_ was to remove electrons from the surface of Au after DH had occurred. More recently, Surendranath and co-workers confirmed that DH can be limited by this process and demonstrated that aerobic alcohol oxidation could be considered as two coupled electrochemical half-cells.^16^ This led to the development of a short-circuit electrochemical model, capable of predicting thermocatalytic performance for a range of substrates across a broad range of conditions.

Confirmation that the ORR limits the rate of alcohol DH is an important observation, which could, and indeed should, have a significant impact on catalyst design. Recently, we demonstrated that a catalyst possessing phase-separated Au and Pd particles was exceptionally active for the DH of several alcohol and formyl species and was, remarkably, more active than an analogous AuPd alloy catalyst.^17^ The origin of this activity was attributed to the coupling of monometallic Au and Pd active sites, where electrons generated through DH on Au sites are transferred to Pd sites and consumed in an ORR. Strikingly, it was demonstrated that these enhancements were observed over physical mixtures of monometallic components, indicating that electron transfer is facilitated through the physical contact of support grains. Cooperative redox enhancement (CORE), the term assigned to this complementary effect, should not be confused with conventional synergy. From a catalyst design perspective, given that the two active components catalyze different reactions, it provides an additional element of control. In our first publication on this topic, all of the testing was conducted over catalysts possessing excess Au (Au/Pd = 4 mol/mol). More recently, we demonstrated that the molar ratio of Au and Pd could also influence CORE.^18^ In this follow-up study, different quantities of the same 1 wt % Au and Pd catalysts were used to study the CORE effects exhibited by physical mixtures in aerobic oxidation reactions. CORE enhancements were observed across a broad ratio of Au and Pd, thus evidencing the generality of the effect. Notably, the magnitude of the effect changed as a function of the Au and Pd ratio. With this in mind, we set out to investigate it in more depth; we altered the weight loadings of Au and Pd in each of the monometallic catalysts and extended the scope to include analogous Au–Pd/C alloy catalysts and phase-separated Au@Pd/C catalysts. This approach not only provided further understanding into the influence of the Au/Pd ratio, but it allowed us to study how physical properties such as particle size also influence CORE.

**Scheme 1:**
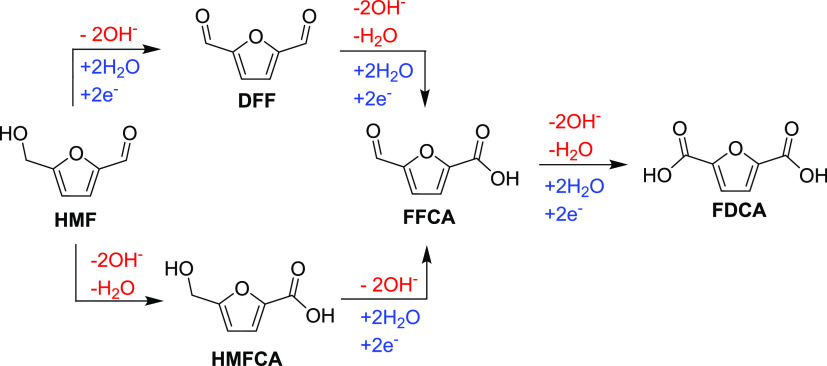
Aerobic Catalytic Oxidation of 5-Hydroxymethylfurfural to Furan Dicarboxylic Acid Proceeds through Several Intermediates 5-Hydroxymethylfurfural (HMF); 5-hydroxymethyl-2-furancarboxylic acid (HMFCA); furan-2,5-dicarbaldehyde (DFF); 5-formyl-2-furancarboxylic acid (FFCA); furan-2,5-dicarboxylic acid (FDCA).

Although we previously demonstrated that CORE effects are observed across a broad range of substrates,^17^ 5-hydroxymethylfurfural (HMF) was considered the most suitable substrate to continue our investigations (Scheme 1). In addition to ensuring consistency across our previous works^17,18^ and providing us with a tool to compare our results with the expansive library of associated literature,^19,20^ the presence of both alcohol and formyl moieties makes it an interesting substrate to study selective oxidation chemistry and the terminal product, furan-2,5-dicarboxylic acid (FDCA), is of commercial interest.^21,22^

## Experimental Procedures

### Main Chemicals (Source, Purity)

Chloroauric acid (Strem Chemicals, 99.8%); palladium chloride (Sigma-Aldrich, >99.9%); poly(vinyl alcohol) (Sigma-Aldrich, *M*_w_ 9000–10,000, 80% hydrolyzed); sodium borohydride (Sigma-Aldrich, 99.99%); 5-hydroxymethyl-2-furancarboxylic acid (Carbosynth, >97.0%); distilled water millipore (18.2 MΩ·cm at 25 °C); 5-hydroxymethylfurfural (Sigma-Aldrich, >99.0%); 5-formyl-2-furancarboxylic acid (Fluorochem); 2,5-furandicarboxylic acid (Sigma-Aldrich, 97%); molecular O_2_ (BOC, >99.95%); Nafion (Sigma-Aldrich, 5 wt % in lower aliphatic alcohols and water, contains 15–20% water); sodium hydrogen carbonate (Fisher Scientific, >99.5%); sodium hydroxide (Fisher Scientific); Carbon Vulcan XC-72R (Cabot Corporation); ABTS (2,2′-azinobis [3-ethylbenzothiazoline-6-sulfonic acid]-diammonium salt) (Sigma-Aldrich, ≥98%); horseradish peroxidase (Sigma-Aldrich, 141.9 U/mg solid); hydrogen peroxide (Fisher Scientific, 30 wt %).

### Catalyst Preparation

All of the catalysts used in this study were prepared using an identical sol-immobilization methodology as outlined in our previous paper.^17^ It is important to note that the quantity of metal precursors (HAuCl_4_·3H_2_O and PdCl_2_) used in the preparation of each catalyst changed depending on the target metal loading of each catalyst.

#### Preparation of the Monometallic Au/C and Pd/C Catalysts

For the synthesis of monometallic Au/C or Pd/C catalysts, desired quantities of HAuCl_4_ solution (9.55 mg mL^–1^) or PdCl_2_ solution (10 mg mL^–1^) and a magnetic stirrer were added to a beaker containing deionized (DI) water (140 mL) and stirred continuously. To this, the required amount of poly(vinyl alcohol) (PVA) (PVA/metal (w/w) = 1/1) was added and the solution was left to stir for a further 5 min. Subsequently, the desired quantity of a freshly prepared NaBH_4_ solution (0.15 M, NaBH_4_/metal (mol/mol) = 4/1) stored in an ice bath, was added, leading to a sudden color change; a dark red color signified the formation of a Au colloid, and a dark brown color signified the formation of a Pd colloid. After a further 30 min of stirring, the desired quantity of Vulcan XC-72R carbon support was added (typically 0.5 g). After 30 min of vigorous stirring, the slurry was filtered and washed thoroughly with 1 L of DI water. The resulting sample was then transferred to dry in an oven (110 °C) for 16 h.

#### Preparation of the Au–Pd Alloy Catalyst (Au–Pd/C)

The AuPd alloy catalysts were prepared using a similar method to that described above. Briefly, HAuCl_4_, PdCl_2_, and PVA were added to 140 mL of DI water. The metal salts were subsequently reduced by the instantaneous addition of NaBH_4_ leading to the formation of a dark brown colloid. After 30 min of stirring, the desired amount of Vulcan XC-72R carbon was then added to the colloidal solution and after a further 30 min of stirring, the slurry was filtered, washed with DI water (1 L), and dried in an oven (110 °C) for 16 h.

#### Preparation of the Phase-Separated Binary Mixture (BM) Catalysts (Au@Pd/C)

Once again, a similar methodology to that utilized above was adopted for the synthesis of these materials. The primary difference between the preparation of these materials and equivalent alloy catalysts is that the Au and Pd colloids were generated separately. They were then combined into a larger beaker and the XC-72R support was added immediately. After 30 min of further stirring, the slurry was again filtered, washed with DI water (1 L), and dried in an oven (110 °C) for 16 h.

### Catalyst Testing

#### Thermocatalytic Experiments

Aqueous-phase aerobic HMF oxidation was conducted in a glass Colaver reactor (50 mL). In a typical reaction, a certain amount of catalyst (71.5 and 143.1 mg of monometallic and bimetallic catalysts, separately) was added into 16 mL of an aqueous solution containing HMF (0.1 M) and NaHCO_3_ (0.4 M). The reactor was then purged and charged with O_2_ (3 barG), sealed, and immersed in an oil bath stabilized at 80 °C. The gaseous reagent was continuously supplied to the reactor. Over the course of the experiments, the mixture was stirred continuously (1000 rpm). At specific time intervals (typically, 5, 15, 30, 60, and 90 min) the reactor was depressurized, and liquid samples (0.2 mL) were removed. After each sample was taken, the reactor was rapidly re-purged (3 times) and re-charged with O_2_ (3 barG). Each aliquot was subsequently diluted in DI water (30-fold) and centrifuged to remove any residual catalyst present prior to analysis.

Reaction components in each sample were separated and analyzed on a high-performance liquid chromatography instrument (Agilent Technologies 1200 series), equipped with a Hi-Plex H column (300 mm × 7.7 mm) and a diode array detector (DAD). A dilute solution of H_2_SO_4_ (5 mM) was used as the mobile phase for this analysis, which flowed at a constant rate (0.7 mg mL^–1^) for the duration of the method. Quantification of reaction components was achieved through comparison with external calibrations. A wavelength of 254 nm was used to monitor each component using the DAD.

HMF conversion, product selectivity, product yield, carbon balance, and reaction rate were determined using eqs 1–5. Equation 6 was used to quantify activity, taking into account sequential turnovers (activity_STO_).
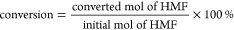
1
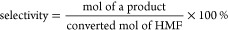
2
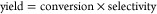
3
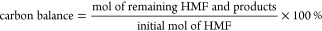
4
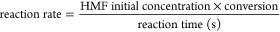
5
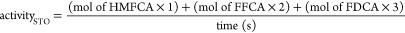
6Hydrogen peroxide generated during the reaction was quantified using a colormetric method with an Agilent Cary 60 UV–vis spectrophotometer. In a typical test, 0.2 mL of filtered reaction solution was added to 0.8 mL of 1 mM ABTS solution (phosphate buffer, pH = 6) in an ice bath. 10 μL of 354 U mL^–1^ HRP (horseradish peroxidase) was added to the above mixture prior to the UV–vis test to yield a green end product if there is any H_2_O_2_ in the reaction solution. Absorbance was measured at 651 nm. A calibration function curve of absorbance vs H_2_O_2_ concentration was obtained by diluting a standard mixture of 30 wt % H_2_O_2_.

#### Electrocatalytic Experiments

##### Preparation of the Working Electrode

Monometallic (7 mg) or bimetallic (14 mg) catalysts were dispersed in DI water (1 mL) and Nafion solution (0.1 mL) to form an ink. The resulting ink was sonicated for 150 s to ensure uniformity. The ink (0.02 mL) was subsequently deposited on polished and acid-cleaned glassy-carbon electrode with a working area of 0.07065 cm^2^. The electrode was left to dry at room temperature for 16 h.

##### Half-Cell Experiments

The electrochemical experiments were performed in a homemade glass reactor with a three-electrode setup controlled by a potentiostat (BioLogic Sciences Instruments Ltd.). A glassy-carbon electrode was used as the working electrode (WE), a Pt coil (BASinc, 7.5 cm long and 0.5 mm diameter, 99.95% purity) was used as the counter electrode (CE) and a saturated calomel electrode (RE-2BP, ALS, Japan) was used as the reference electrode (RE). Cyclic voltammetry (CV) experiments were performed with nitrogen bubbling continuously (150 mL min^–1^) through a NaOH electrolyte solution (45 mL, 0.1 M) for 20 min to remove any remaining oxygen, and the system was then left under nitrogen atmosphere, at ambient pressure, during the reaction. The WE was pre-reduced at a negative potential for 10 s and a background was then recorded for three cycles ranging from 0.2 to 1.4 V vs RHE. HMF solution (5 mL, 0.2 M) was subsequently added into the NaOH solution and CV traces were measured for another 3 cycles at the same scanning rate and over the same range. All of the CV experiments were conducted at a constant sweep rate of 50 mV s^–1^. Additionally, ORR was monitored through linear sweep voltammetry (LSV) with oxygen bubbling (50 mL min^–1^) for 30 min beforehand to saturate the solution. These experiments were performed with and without HMF (0.02 M), ranging from 1.2 to 0.4 V vs RHE. All of the current densities reported are normalized to the surface area of the working electrode (0.07065 cm^2^).

##### Dual-Cell Experiments

The dual chamber cell was separated by an anionic membrane (HMED-0510-2, HUAMOTECH, China). The current was measured by a potentiostat with no potential applied to the system. In a typical experiment, 31.5 mL of 0.1 M NaOH was added to both compartments. Oxygen was bubbling in one compartment and nitrogen in the other compartment for 20 min with a flow rate of 150 mL min^–1^. HMF (3.5 mL, 0.2 M) was added to both compartments. While the oxygen was bubbling, the Au/C electrode was rapidly introduced into the oxygen cell and the Pd/C electrode into the nitrogen cell. The flow rate of each gas was reduced to 50 mL min^–1^ before the connection of the electrodes. The current was recorded using a potentiostat after the initial catalysts wetting and activation period was complete. The same concentration of electrolyte (0.1 M NaOH) was used in both cells to avoid any diffusion between individual components of the dual cell.

Note: The concentration of NaOH used in the electrocatalytic experiments was significantly higher than that used in the thermocatalytic experiments (*ca.* pH 14 vs pH 8.7). A higher pH was used in the electrocatalytic experiments as it was required to acquire statistically relevant data. The experimental conditions used herein were acquired from a previous publication that investigated the electrocatalytic oxidation of HMF.^17^Figure S1 shows that only limited current density is observed when electrochemical experiments are conducted at the same pH as the thermocatalytic experiments. Running the thermocatalytic experiments at high pH provides a further complication as this can promote bimolecular reactions, the products of which can result in catalyst deactivation.^23^

The reaction mechanism observed in the present manuscript is the same as the one described in our previous paper.^17^ However, the conversion and the selectivity observed are different, due to differences in the batch of Vulcan XC72-R and HAuCl_4_·3H_2_O stock solution used to prepare the catalysts in the present study.

### Catalyst Characterization

#### Transmission Electron Microscopy (TEM)

Micrographs of the monometallic Au/C and Pd/C catalysts were acquired using a JEOL 2100-JEM instrument operated at 200 kV. Samples were prepared by dry dispersing method onto 300-mesh copper grids coated with holey carbon film Particle size distributions for each of these catalysts were determined through analysis of particles within these micrographs using Fiji software (minimum 300 particles count).

#### Scanning Transmission Electron Microscopy (STEM) and Energy-Dispersive X-ray Analysis (STEM-EDX)

Micrographs and analysis of Au*_x_*@Pd*_y_*/C catalysts were acquired using STEM high-angle annular dark-field (HAADF) imaging, operated on an aberration-corrected JEOL-ARM200F microscope equipped with a cold-field emission gun and an Oxford Instrument X-ray Energy-Dispersive Spectrometer.

#### X-ray Photoelectron Spectroscopy (XPS)

XPS measurements were performed using a Thermo Scientific K-Alpha+ system which utilizes micro-focused Al Kα radiation operating at a power of 72 W (6 mA × 12 kV). The system uses a combined low-energy electron–low-energy ion source for charge compensation. Unless otherwise specified, all data were collected at a pass energy of 40 eV with a 0.1 eV step size, using the 400 μm spot mode, which is an elliptical area of approximately 400 μm × 600 μm and defines the analysis area. Data were analyzed using CasaXPS v2.3.25.^24^ Where required data was calibrated to the C 1s peak for aliphatic C–C/C–H bonds, taken to be 285 eV, and quantified after subtraction of a Shirley-type background. For the K-Alpha+ system, atomic ratios were calculated using Scofield sensitivity factors^25^ with an electron escape depth correction according to the TPP-2M formula of Tanuma et al.^26^ as recommended by the instrument manufacturer. Where fitting was required, asymmetric and Voigt-type functions were used according to the LA line shape in CasaXPS and derived from analysis of bulk samples.

## Results and Discussion

To begin our investigation, a series of monometallic Au*_x_*/C and Pd*_y_*/C catalysts, AuPd alloy catalysts (Au*_x_*–Pd*_y_*/C), and catalysts composed of phase-separated monometallic Au and Pd particles (Au*_x_*@Pd*_y_*/C) were synthesized by sol-immobilization. The nomenclature assigned to these catalysts, their theoretical weight loadings, and mean supported metal particle sizes (based on micrographs displayed in Figures S2 and S3) are presented in Table S1. In all cases, subscript values “*x*” and “*y*” correspond to the molar percent of Au and Pd present. The loading of Au and/or Pd was adjusted to ensure that the total mass of carbon was kept constant in reactions where they were employed as a physical mixture.

**Figure 1:**
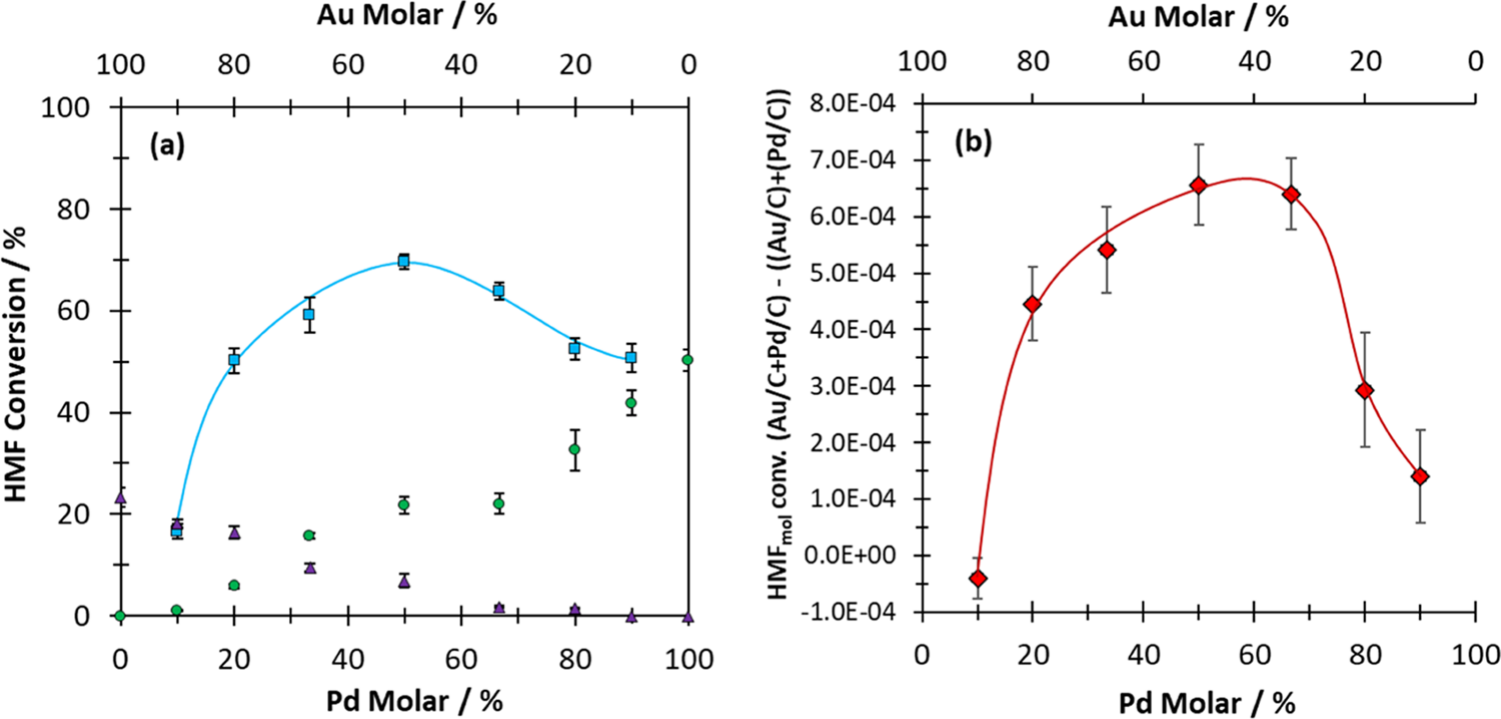
(a) Influence of the Au and Pd molar ratio on HMF conversion over monometallic Au/C (purple triangles), Pd/C (green circles), and a physical mixture of the two components (blue squares). (b) Difference in moles of HMF converted in reactions where Au*_x_*/C and Pd*_y_*/C are reacted as a physical mixture is compared against the sum of HMF moles converted in independent reactions over monometallic equivalents. Associated error bars correspond to mean ± s.d.(*n* = 3). Reaction conditions: 0.1 M HMF; 0.4 M NaHCO_3_; 16 mL of H_2_O; 80 °C; pO_2_ = 3 bar; 30 min.

First, the activity of the monometallic catalysts was compared to their activity as a physical mixture. The quantity of HMF converted, after 30 min of reaction, over the various monometallic catalysts and physical mixtures is displayed in Figure 1a. Supplemental activity and yield data for these experiments are listed in Tables S2–S4. For reactions over monometallic Au*_x_*/C and Pd*_y_*/C catalysts, HMF conversion increased as the quantity of each metal present in the reaction was increased. An increase in the HMF conversion was observed when Au/C and Pd/C were reacted as a physical mixture. Notably, this was the case across a broad Au/Pd ratio (Figure 1b). This is an important observation, as it demonstrates that CORE is not limited to Au-rich systems; the sole region investigated in our previous work.^17^ Interestingly, the extent of the CORE effect appears to be influenced by the Au/Pd ratio. The optimum CORE effect, that is, the greatest difference between the sum of HMF converted over a physical mixture (Au*_x_*/C + Pd*_y_*/C) and that observed over separate components was seen when equimolar quantities of Au_50_/C and Pd_50_/C catalysts were used. In this regime, the sum of HMF converted over the Au_50_/C catalyst and (separately) the Pd_50_/C catalyst equalled 28.6%. This was significantly lower than the HMF conversion observed in the analogous reaction, where identical quantities of Au_50_/C and Pd_50_/C were reacted as a physical mixture (69.7%). This equates to 2.4 times increase in HMF conversion, which is superior to the enhancement reported in our previous work (2.0 times increase) conducted under identical reaction conditions with an Au-rich/Pd-lean catalyst formulation (Au_80_/C and Pd_20_/C).

Given that the particle size of supported metal catalysts is known to affect the rates of both ODH^27−29^ and the ORR,^30−33^ it was important to assess whether this parameter was influenced by metal loading. Representative micrographs and particle size distributions (PSD) obtained by TEM analysis for a selection of monometallic Au*_x_*/C and Pd*_y_*/C catalysts are presented in Figures S2 and S3, respectively. Interestingly, increasing the loading of Au appeared to have very little influence on particle size. Despite the broad range of weight loadings, from 0.62 (Au_20_/C) to 1.75 wt % (Au_80_/C), the mean particle sizes were relatively consistent; all were found to possess a mean particle size of between 2.7 and 2.8 nm, with the exception of the Au_80_/C catalyst (mean particle size = 3.2 nm). This was not true for the series of Pd*_x_*/C catalysts, where a fairly linear increase in Pd particle size (from 1.6 to 2.3 nm) was observed when the Pd loading was increased from 0.24 wt % (Pd_20_/C) to 1.35 wt % (Pd_80_/C). It is important to note that there may be limitations associated with these measurements, due to instrument resolution constraints. Thus, small clusters (<1 nm in diameter) are not likely to be accounted for in these measurements. This hypothesis is supported by the large standard deviation associated with the particle size distributions (σ = 0.9–1.2 nm).

**Figure 2:**
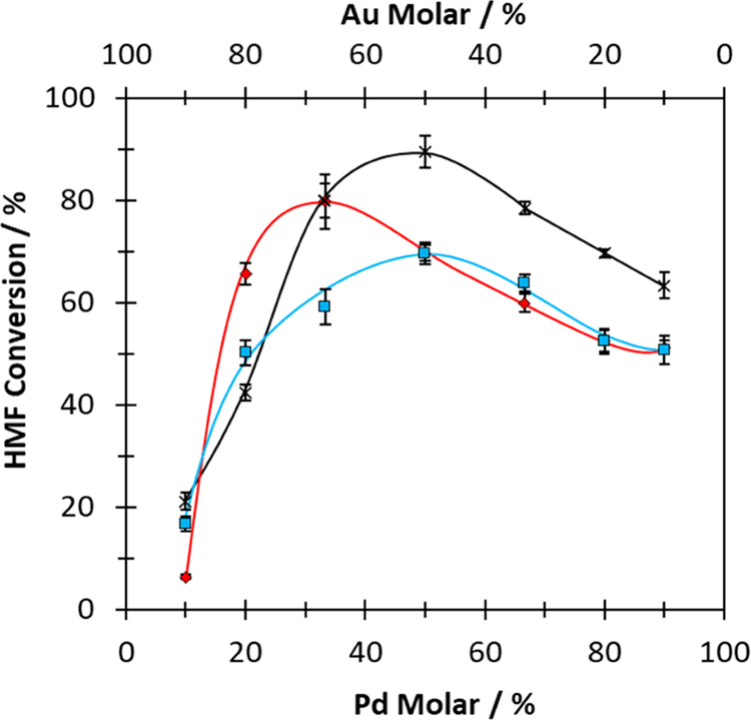
Influence of the Au and Pd molar ratio on HMF conversion over a range of bimetallic Au and Pd systems. KEY: Physical mixture of monometallic Au/C and Pd/C (blue squares); Au@Pd/C catalyst (red diamonds); Au–Pd/C alloy catalyst (black crosses). Associated error bars correspond to mean ± s.d.(*n* = 3). Reaction conditions: 0.1 M HMF; 0.4 M NaHCO_3_; 16 mL of H_2_O; 80 °C; pO_2_ = 3 bar; 30 min.

To assess the significance of the CORE effect observed over the physical mixtures, an analogous series of phase-separated (Au*_x_*@Pd*_y_*/C) and alloyed (Au*_x_*–Pd*_y_*/C) catalysts were synthesized and evaluated for HMF oxidation, under identical conditions. The HMF conversion data from these experiments are presented in Figure 2, with supplemental activity and yield data reported in Tables S5 and S6. With the series of alloyed Au*_x_*–Pd*_y_*/C catalysts, the highest HMF conversion was exhibited by the Au_50_–Pd_50_/C catalyst.

**Figure 3:**
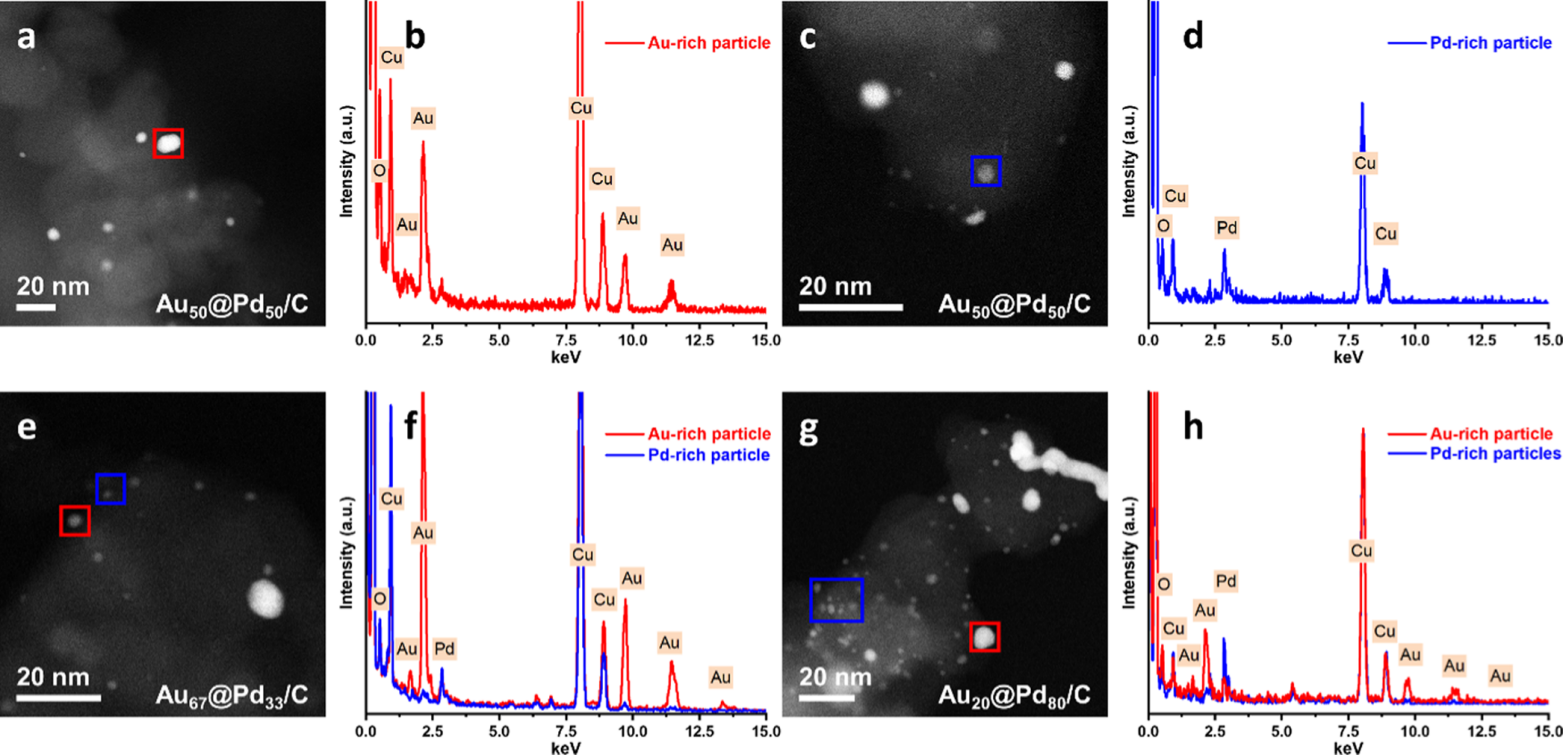
STEM-EDS data of Au@Pd/C catalysts: (a–d) Au_50_@Pd_50/_C; (e, f) Au_67_@Pd_33_/C; and (g, h) Au_20_@Pd_80/_C.

Notably, this was the same optimum Au/Pd ratio observed in reactions over physical mixtures of Au*_x_*/C and Pd*_y_*/C. The alloyed Au_50_–Pd_50_/C catalyst was however more active, exhibiting an HMF conversion of 89.5% after 30 min of reaction, which corresponded to an activity_STO_ of 14.2 × 10^–7^ mol s^–1^. Interestingly, over the Au*_x_*@Pd*_y_*/C catalysts, the optimum Au/Pd ratio shifts. Over these catalysts, the highest activity was exhibited by an Au-rich formulation; the Au_67_@Pd_33_/C catalyst was determined to be the most active (HMF conversion = 79.9%; activity_STO_ = 12.6 × 10^–7^ mol s^–1^). Selected Au*_x_*@Pd*_y_*/C samples were subsequently characterized by STEM-EDS. Representative micrographs of the Au_20_@Pd_80_/C, Au_50_@Pd_50_/C, and Au_67_@Pd_33_/C catalysts are presented in Figure 3. Evidently, the methodology used for the synthesis of these catalysts had been successful; separate Au-rich and Pd-rich particles were determined to be present in each of the samples. This observation is supported by complementary XPS data; four Au*_x_*–Pd*_y_*/C catalysts and four Au*_x_*@Pd*_y_*/C catalysts, with different Au and Pd ratios (Au/Pd = 9, 4, 1, 0.2), were examined. Inspection of the Au 4f region (Figure S4) confirmed that Au was present in its metallic state in all of the samples. Overlapping peaks from the carbon support made the deconvolution of Pd oxidation states challenging (Figure S5). Thus, we can only confidently state that the catalyst possesses some Pd^0^. Further insights were however acquired by comparing the binding energy of the Au^0^ 4f_7/2_ peak in each series. With the Au*_x_*–Pd*_y_*/C catalysts, the binding energy of the Au^0^ 4f_7/2_ signal shifted lower with the introduction of increasing amounts of Pd, which we attribute to significant alloying. By comparison, the binding energy of the Au^0^ 4f_7/2_ peak remained constant (*ca.* 84.2 eV) for the Au@Pd/C catalysts regardless of the Au/Pd ratio, which is indicative of a lack of alloying in the case of these materials and in keeping with the STEM-EDS studies.

Earlier work has demonstrated that pH can drastically influence reaction rates in aerobic alcohol oxidation.^3^ As such, it was important to assess whether the different activity exhibited by the catalytic systems studied here was a result of pH changes during the reactions. To assess whether this was the case, a series of additional experiments were conducted over the series of catalysts that possessed equimolar quantities of Au and Pd (Au_mol_/Pd_mol_ = 1) and the pH was monitored over time. Over each of the catalysts, only small changes in pH were observed (Table S7), indicating that the differences in performance could not simply be attributed to a pH effect.

To understand why the Au*_x_*/C + Pd*_y_*/C and Au*_x_*@Pd*_y_*/C catalytic series exhibited activity optima at different Au/Pd ratios (1 and 2, respectively), the origin of the CORE effects must be considered. In our earlier work, we confirmed that, over physical mixtures of Au/C and Pd/C, the rate of DH which occurs at Au sites is limited by the ORR taking place at coupled Pd sites. Recall that both Au and Pd can (independently) catalyze HMF DH (Figure 1) and ORR. In thermocatalytic reactions over the bimetallic catalysts, it can be considered that these reactions all occur simultaneously and are in competition. In this case, the magnitude of the CORE effect is therefore likely to be dependent on the associated rate constants, and the ease by which electrons can be transferred about the system (or from one component to the other).

From Figures 1 and 2, it is evident that the maximum in HMF conversion (*m*_hmf_) for the Au*_x_*/C + Pd*_y_*/C, Au*_x_*–Pd*_y_*/C, and Au*_x_*@Pd*_y_*/C series occurs at different molar ratios of Au and Pd. With the Au*_x_*/C + Pd*_y_*/C and Au*_x_*–Pd*_y_*/C series, *m*_hmf_ is observed when equimolar quantities of Au and Pd are present. However, when the contribution from monometallic Au*_x_*/C and Pd*_y_*/C are considered (Figure 1b), *m*_hmf_ for the Au*_x_*/C + Pd*_y_*/C series may occur when there is a slight excess of Pd present. On the contrary, the *m*_hmf_ for the Au*_x_*@Pd*_y_*/C series is clearly exhibited when there is an excess of Au present. The different *m*_hmf_ values observed over these catalysts suggest that there may be multiple CORE mechanisms at play, which are dependent on the catalyst(s) present. It is known that oxygen reduction can proceed through both two-electron and four-electron processes. Over supported Pd catalysts, a four-electron process (O_2_ + 4e^–^ + 4H^+^ → 2H_2_O) is dominant,^34^ while a two-electron process (O_2_ + 2e^–^ + 2H^+^ → H_2_O_2_) is typically favored over supported Au catalysts.^35^ This is further evidenced by the fact that Ketchie et al.^36^ previously observed that H_2_O_2_ is produced as a byproduct in the aerobic aqueous-phase oxidation of glycerol of supported Au catalysts.

Over supported Au catalysts, H_2_O_2_ is relatively stable, but in the presence of Pd, it can rapidly reduce (H_2_O_2_ + 2e^–^ + 2H^+^ → 2H_2_O). To assess whether this was the case, a series of additional experiments were conducted and the concentration of H_2_O_2_ present post-reaction was determined (Table S8). It is important to note that as H_2_O_2_ is inherently unstable under alkaline conditions and at elevated temperatures,^37,38^ the acquired data should only be considered qualitatively. In a thermocatalytic HMF oxidation reaction over the Au_80_/C catalyst, a notable quantity (*ca.* 28 ppm) of H_2_O_2_ was produced. Interestingly, when combined in a reaction with Pd_20_/C, no H_2_O_2_ was observed. This could indicate that in a Au_80_/C + Pd_20_/C coupled system, either: (i) the H_2_O_2_ generated by the Au_80_/C catalyst is reduced by the Pd_20_/C catalyst or (ii) the Au_80_/C does not catalyze the ORR in reactions where both these components are present. Notably, no H_2_O_2_ was observed in reactions over monometallic Au_20_/C, Pd_20_/C, and Pd_80_/C catalysts or in a reaction over a physical mixture of Au_20_/C + Pd_80_/C. Given the proximity of Au and Pd components in the Au*_x_*@Pd*_y_*/C catalysts (Figure 3), it is logical to consider that H_2_O_2_ reduction may be prevalent. Over physical mixtures of Au/C + Pd/C, this reaction is likely to be less dominant, as (i) four-electron processes are dominant on Pd/C catalysts and (ii) any H_2_O_2_ produced through a two-electron process on Au would need to desorb from the Au/C, diffuse through the reaction medium and re-adsorb onto a Pd site on a separate grain of support. Given the inherent instability of H_2_O_2_ at high pH and elevated temperatures, only a marginal CORE effect, arising from H_2_O_2_ reduction, is likely to be observable over Au/C and Pd/C physical mixtures as this process would compete with the conventional DH-ORR coupled process. That said, the asymmetric shape of the CORE effect observed when contributions from monometallic Au/C and Pd/C reactions are deducted (Figure 1b) suggests that some CORE effect, albeit minor, arises from coupled H_2_O_2_ reduction in Au*_x_*/C + Pd*_y_*/C systems. Further work is however required to confirm this hypothesis.

This fundamental understanding may also explain why the performance of the Au*_x_*@Pd*_y_*/C and Au*_x_*/C + Pd*_y_*/C catalysts is almost identical in the Pd-rich region (Au_mol_/Pd_mol_ ≤ 1). HMF DH over both catalyst series must be limited in the same way. Thus, H_2_O_2_ reduction on Pd no longer appears to promote HMF DH. For this reason and the fact that there is more Pd present in this region, it is assumed that here, HMF DH is favored on Pd sites rather than Au. Notably, in this region, both the bimetallic series which exhibit CORE effects were less active than the corresponding Au*_x_*–Pd*_y_*/C catalysts.

Through consideration of the coupled reactions, that is: the two oxygen reduction reactions and H_2_O_2_ reduction, the different *m*_hmf_ observed over the different bimetallic systems can be explained. Over monometallic Au catalysts, where it is known from previous work that DH and ORR proceed simultaneously,^15^ HMF conversion appears to have a square dependency. Where *f* corresponds to the mol fraction of Au present, HMF conversion (HMF_con_) = *f*^2^, yielding *m*_hmf_ at *f* = 1. Over the Au*_x_*@Pd*_y_*/C catalyst series, where *m*_hmf_ occurs at a Au_mol_/Pd_mol_ ratio of 0.67, HMF_con_ = *f*^2^(1 – *f*). Finally, the *m*_hmf_ exhibited by the Au*_x_*/C + Pd*_y_*/C physical mixture, which occurs at a Au_mol_/Pd_mol_ ratio of 0.5, can be described by HMF_con_ = *f*(1 – *f*). Such models cannot be applied to the Au*_x_*–Pd*_y_*/C series, as here HMF ODH is driven by conventional synergy rather than CORE effects.

To gain greater insight into the similar behavior of the Au*_x_*/C + Pd*_y_*/C and Au*_x_*@Pd*_y_*/C series in the Pd-rich region (Au_mol_/Pd_mol_ ≤ 1), the conversion of HMF over selected catalysts was monitored over time (Figure S6). Evidently, the Pd_50_/C and Pd_80_/C monometallic catalysts are prone to deactivation; an effect that is more pronounced over the Pd_80_/C catalyst. Notably, all of the other catalysts with this Au/Pd ratio (Au_mol_/Pd_mol_ = 0.25), except the monometallic Au_20_/C catalyst, also exhibited deactivation, but to a lesser degree. The deactivation of supported Pd catalysts in alcohol oxidation is well documented in the literature, with researchers having predominantly attributed this to irreversible Pd oxidation or product inhibition.^39,40^ To assess whether product inhibition was responsible for the deactivation of Pd in this work, subsequent HMF oxidation experiments were conducted in the presence of FFCA (HMF_mol_/FFCA_mol_ = 1) (Figure S7). HMF conversion over Au_80_/C, Au_50_–Pd_50_/C, and Au_20_–Pd_80_/C appeared to be unaffected by the presence of FFCA. In contrast, significant inhibition was observed over the Pd_80_/C catalyst; HMF conversion dropped from 12.9 to 8.1% after 5 min of reaction, in the presence of excess FFCA. We have confirmed that the Au and Pd components are, for the most part (Figure 3), phase-separated in the Au_20_@Pd_80_/C and have previously ruled out metal migration in similar physical mixture systems.^17^ Thus, it is likely that the Pd sites present in these catalysts also experience product inhibition. Indeed, product inhibition from other intermediates could also be likely. Evidently, alloying Pd with Au (Au_20_–Pd_80_/C) appears to reduce this effect; an observation that has been noted by several groups previously.^4,41,42^ We therefore attribute the higher performance of the Au–Pd/C catalysts in the Pd-rich region (Au_mol_/Pd_mol_ ≤ 1) to the fact that alloyed Au–Pd particles more effectively suppress product inhibition. To assess whether such deactivation might influence the thermocatalytic reactivity data presented (Figures 1 and 2, and Tables S2–S6), a series of initial rate experiments were conducted over a range of monometallic Au/C and Pd/C catalysts and corresponding physical mixtures, where Au_mol_/Pd_mol_ = 4, 1 and 0.25 (Table S9). Given that, in each case, the magnitude of the CORE effects appeared to be unchanged (within error), we can be confident that the reaction data presented in Figure 1 is accurate.

**Figure 4:**
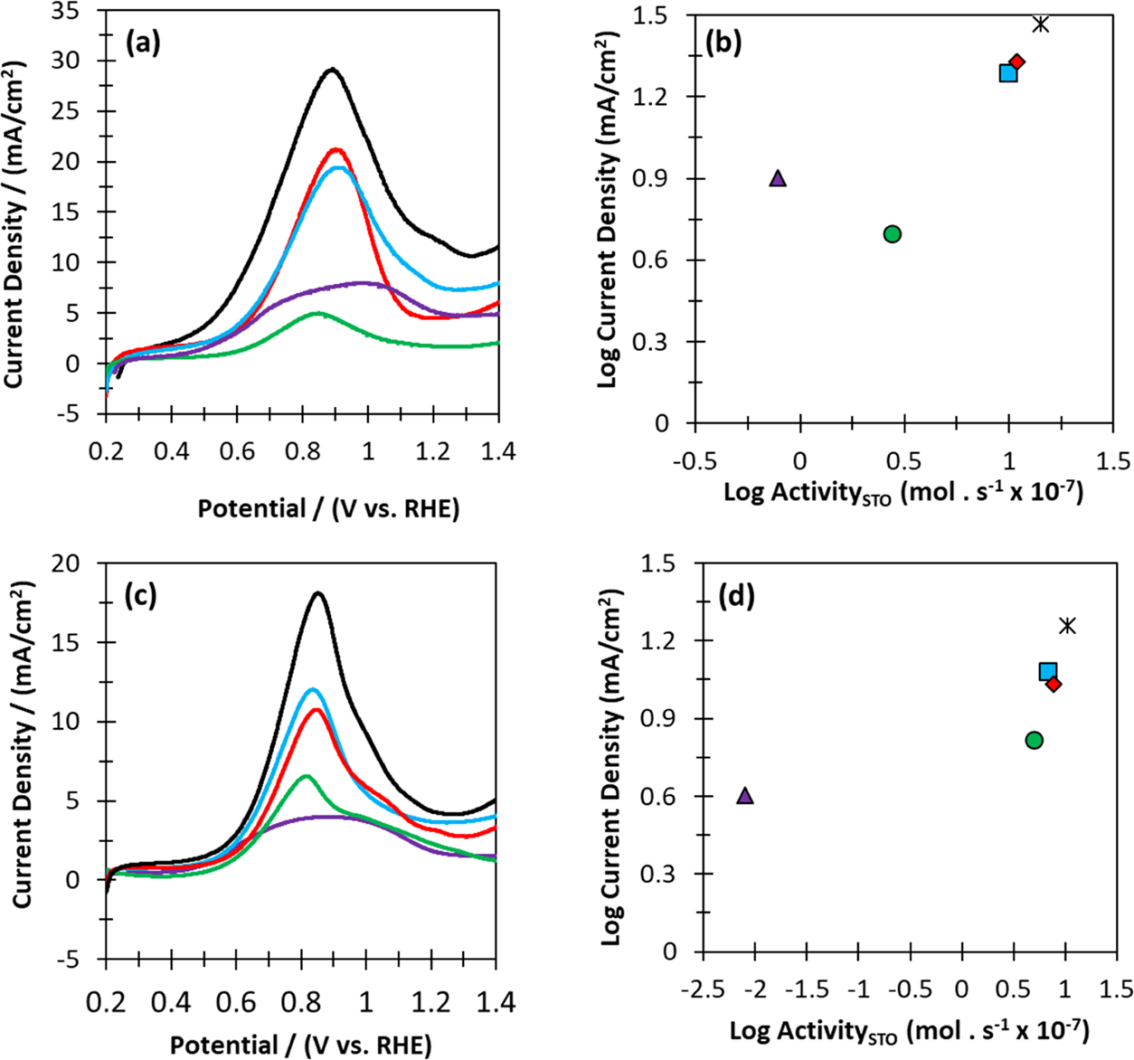
Cyclic voltammetry (anodic scan) of half-cell with HMF solution for catalyst series at the (a) equimolar and (c) Pd-rich regions. The net current density is the difference between anodic-scan current density and blank current density (0.1 M NaOH only). Electrocatalytic reaction conditions: 0.1 M NaOH; 0.02 M HMF; 50 mL of H_2_O; 25 °C; scan rate, 50 mV. s^–1^; N_2_ flow, 50 mL. min^–1^. Thermocatalytic reaction conditions: 0.1 M HMF; 0.4 M NaHCO_3_; 16 mL of H_2_O; 80 °C; pO_2_ = 3 bar; 30 min. LFER plots demonstrating the correlation between thermocatalytic and electrocatalytic HMF oxidation over the catalysts series at the (b) equimolar (Au_50_/Pd_50_) and (d) Pd-rich regions (Au_20_/Pd_80_). KEY: Au/C (purple line/triangles); Pd/C (green line/circles); Au/C + Pd/C (blue line/squares); Au@Pd/C (red line/diamonds); Au–Pd/C (black line/crosses).

To investigate whether the Au/Pd ratio did influence whether HMF DH occurs at Au or Pd sites, two catalyst series; the equimolar series (Au_mol_/Pd_mol_ = 1) and a Pd-rich series (Au_mol_/Pd_mol_ = 0.25), were examined electrochemically. HMF oxidation CV profiles for each of the catalysts are presented in Figure 4a–c. Linear free energy relationship plots (LFER) confirmed that, in general, there was good consistency between the peaks in current density and their thermocatalytic activity, with the Au*_x_*/C catalysts being the only notable outlier (Figure 4b–d). A further understanding was acquired through consideration of the catalysts’ CV onset potentials (Table S10). Interestingly, the onset potentials for the Pd_50_/C, Au_50_/C + Pd_50_/C physical mixture, and the Au_50_@Pd_50_/C catalysts were found to be comparable (0.51–0.53 V). Equally, the onset potentials for the Au_50_–Pd_50_/C alloy and the Au_50_/C were comparable (0.35–0.38 V). Based on these observations, we tentatively propose that when equimolar Au and Pd are present (Au_mol_/Pd_mol_ = 1), HMF DH proceeds at Pd sites.

On the contrary, the onset potentials for the Au_50_/C and the alloy catalysts (Au_50_–Pd_50_/C) were comparable, suggesting that HMF DH is likely to preferentially proceed on Au sites. With the Pd-rich catalyst series (Au_mol_/Pd_mol_ = 0.25), different observations can be made. Here, the onset potentials for the Pd_80_/C, Au_20_–Pd_80_/C, Au_20_@Pd_80_/C and the Au_20_/C + Pd_80_/C catalysts were identical (0.47 V). This suggests that when the Pd: Au ratio is increased, HMF DH also takes place at Pd sites in the alloy catalyst. While only hypothetical at this stage, collectively, these data could indicate that the roles of Au and Pd in coupled systems might be interchangeable.

**Figure 5:**
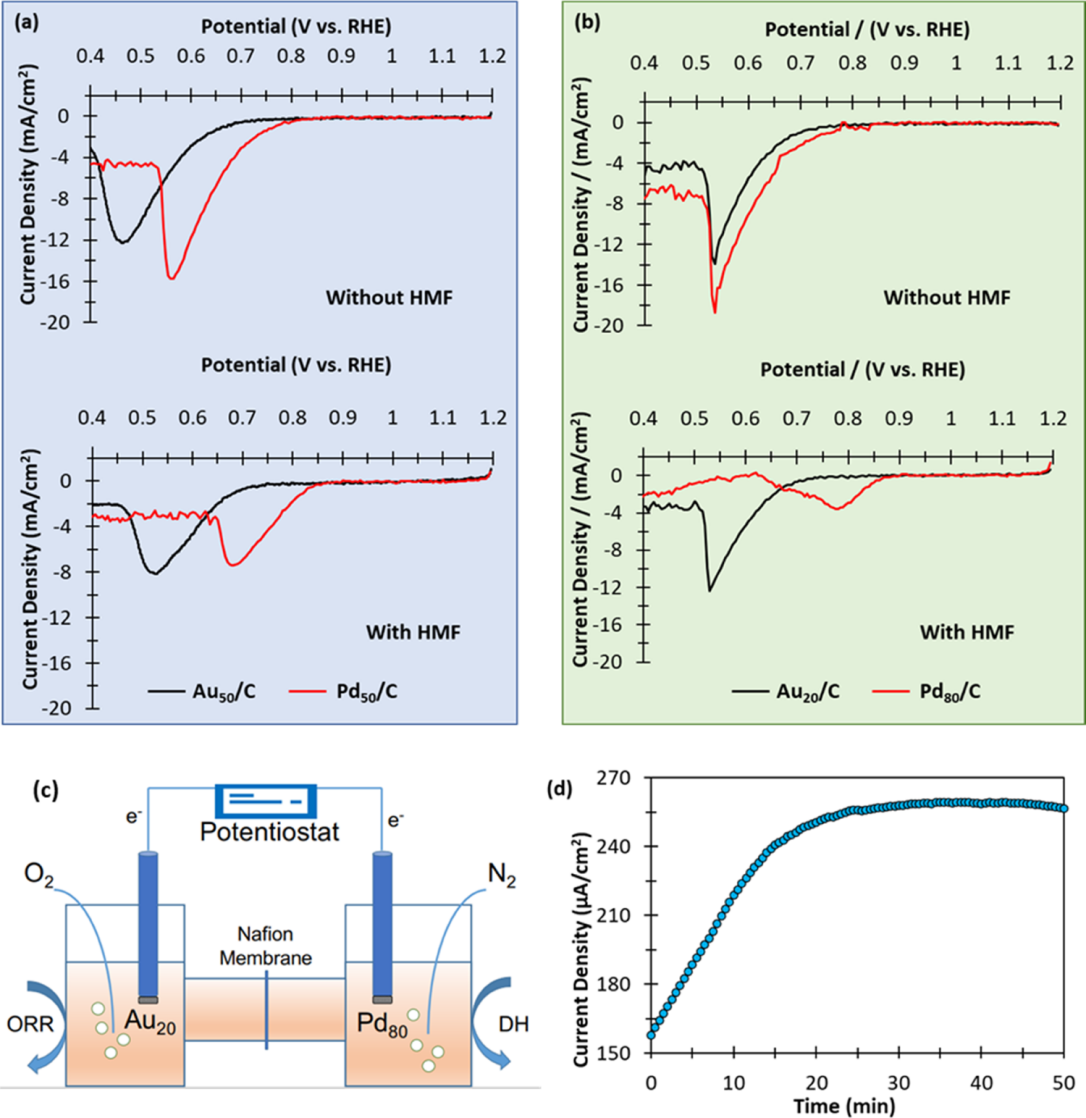
ORR polarization curves for monometallic Au/C and Pd/C catalysts of half-cell in the presence (0.2 M) and absence of HMF solution. Reaction conditions: 0.1 M NaOH; 50 mL of H_2_O; 25 °C; scan rate, 50 mV s^–1^; O_2_ flow, 50 mL min^–1^. ORR is monitored over the monometallic Au/C (black lines) and Pd/C (red lines). (a) Au_50_/C and Pd_50_/C in the absence and presence of HMF; (b) Au_20_/C and Pd_80_/C in the absence and presence of HMF. (c) Schematic representation of dual-cell setup, whereby the anode (Pd/C) is present under N_2_ and the cathode (Au/C) is present under O_2_. (d) Short circuit with current generated as a function of time in a dual H-cell. Reaction conditions: each cell consists of 0.09 M NaOH; 0.02 M HMF; 35 mL of H_2_O; Pd_80_/C (anode under N_2_) or Au_20_/C (cathode under O_2_); 25 °C; O_2_/N_2_ flow, 50 mL min^–1^.

Next, the influence of HMF on ORR over monometallic Au_50_/C, Pd_50_/C, Au_20_/C, and Pd_80_/C catalysts was assessed. Cathodic scans over each of these catalysts, in the presence and absence of HMF, are presented in Figure 5. The presence of HMF appeared to influence the ORR activity (through comparison of onset potentials—Table S11) and current density maxima. Over the Pd*_y_*/C catalysts, the presence of HMF significantly reduced the maximum current density. This can be explained by the competitive adsorption of HMF onto Pd active sites which, logically, would reduce the surface coverages of O_2_ and OH^–^. A similar observation is observed over the analogous Au*_x_*/C catalysts. Interestingly, HMF does not appear to influence the ORR activity over the Au*_x_*/C catalysts; onset potentials of 0.73–0.74 V ± 0.01 were observed for all of the LSV experiments over the Au_50_/C and Au_80_/C catalysts in the presence and absence of HMF. The onset potentials over the Pd*_y_*/C catalysts were comparatively higher (0.78–0.88 V), which is unsurprising given that supported Pd catalysts are known to be more efficient at catalyzing ORR than equivalent supported Au catalysts.^43,44^ Interestingly, the onset potentials for the Pd*_y_*/C catalysts (Pd_50_/C and Pd_80_/C) increased when HMF was present, which we attribute to a particle size effect. From the determination of the mean particle size of the Pd*_y_*/C catalysts (Figure S3), it is clear that the stabilizing nature of the PVA used in the preparation is not completely effective; the particle size increases with Pd loading. We, therefore, propose that the increased ORR activity observed in the presence of HMF is attributed to the ORR taking place on the smaller Pd particles present, while HMF adsorption inhibits the ORR on large particles. This is supported by the significant drop in the current density maxima, which are exhibited by these catalysts in the presence of HMF (Figure 5).

To ensure that electrochemical experiments could be used to provide qualitative insights into the thermocatalytic systems, a series of thermocatalytic experiments were conducted under comparable reaction conditions to the electrochemical experiments (Table S12). CORE effects were observed in reaction over all of the Au/Pd ratios studied, confirming that the insights acquired from the electrochemical experiments could be used for qualitative analysis.

Collectively, the electrochemical experiments provide insight into why the Au/Pd ratio influences the performance of the catalyst series differently. We attribute the similar DH activity of the Au*_x_*/C + Pd*_y_*/C physical mixture and Au*_x_*@Pd*_y_*/C catalysts in the Pd-rich region (Au_mol_/Pd_mol_ ≤ 1) to the fact that here, DH is catalyzed by Pd sites and ORR is catalyzed by Au sites. Given that the rate of ORR over Au catalysts is significantly lower compared to equivalent Pd catalysts, the balance between DH and the ORR in the coupled system is lost. The proximity between Au and Pd is no longer rate-limiting. In the presence of HMF, both the Au_50_/C and Pd_50_/C catalysts still catalyze the ORR, but likely at different rates. LSV measurements confirmed that the presence of HMF influences ORR taking place on Au and Pd sites. Most notably however, the extremely similar cathodic onset potentials exhibited by the Pd-containing catalysts in the Pd-rich region (Pd_80_/C, Au_20_@Pd_80_/C, Au_20_–Pd_80_/C, Au_20_/C + Pd_80_/C) and a prove-of-concept experiment using an H-cell (Figure 5c,d), suggests that HMF DH occurs at Pd sites in these catalysts and generated electrons migrate to Au sites to catalyze ORR. This is in stark contrast to what we observed previously with Au-rich systems^17^ and suggests that the role of metals in CORE systems can be interchangeable, at least in the example presented here. We hypothesize that this interchangeability is attributed essentially to the relative rates of DH, the ORR, and other coupled redox reactions that are taking place. The metal particle size is undoubtedly likely to be a principal factor that influences these reaction rates, which is supported by many previous works that demonstrate particle size and morphology can drastically influence the behavior of supported metal catalysts.^45,46^ Further evidence that particle size effects were influencing the reactivity of the monometallic Au catalysts was subsequently acquired from running HMF oxidation experiments over different masses of the Au_20_/C and Au_80_/C catalysts (Table S13). For the Au_80_/C catalyst, the activity_STO_ approximately doubles, which aligns with the doubling of the catalyst mass used. On the contrary, the Au_20_/C catalysts exhibit an extremely low catalyst performance; almost no activity is observed. These data provide further evidence that the weight loading of the Au*_x_*/C catalysts strongly influences their performance in thermocatalytic HMF oxidation, providing further evidence that this parameter influences the structural properties of the catalysts.

**Figure 6:**
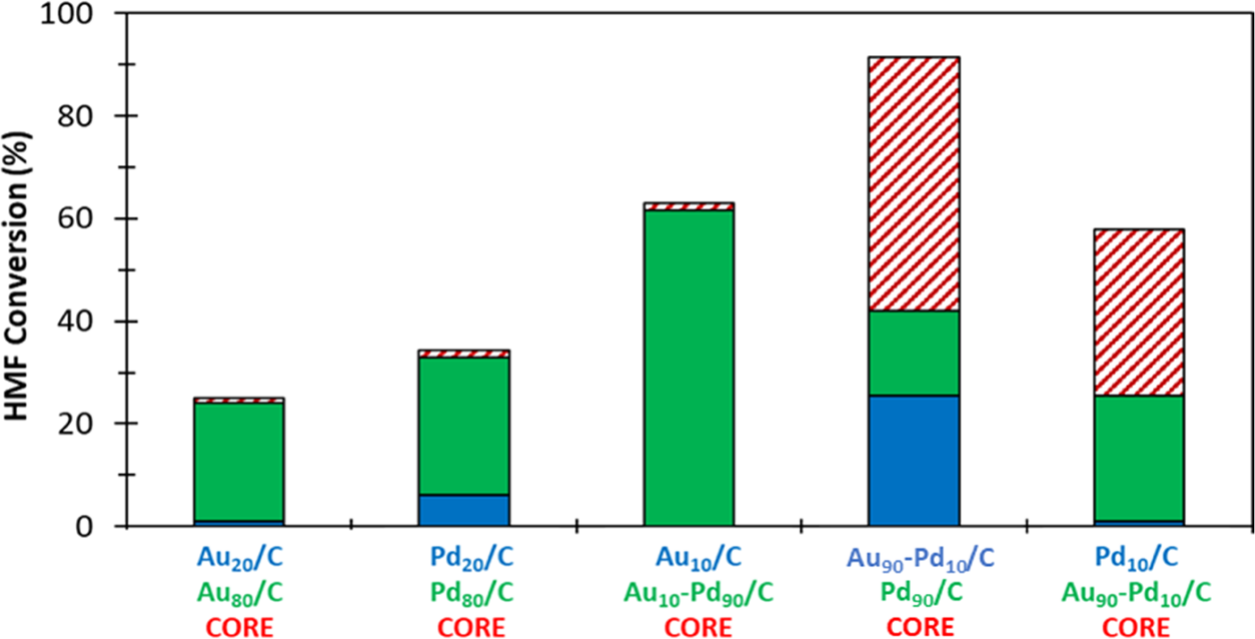
CORE enhancement observed in HMF oxidation over physical mixtures of various combinations of carbon-supported monometallic and alloy catalysts. KEY: Blue and green columns correspond to HMF conversion exhibited by catalysts reacted independently. Red and white column corresponds to the increased HMF conversion (CORE) observed when the two components are reacted as a physical mixture. Reaction conditions: 0.1 M HMF; 0.4 M NaHCO_3_; 16 mL of H_2_O; 80 °C; pO_2_ = 3 bar; 30 min.

To uncover the properties that govern electron transfer between components in physical mixtures, a series of additional thermocatalytic HMF oxidation experiments were conducted over various catalyst combinations. (Figure 6). Interestingly, no CORE was observed when physical mixtures of catalysts possessing the same metal were used (*e.g.*, Au_20_/C + Au_80_/C, Pd_20_/C + Pd_80_/C). Similarly, no CORE was observed when a Pd-rich alloy (Au_10_–Pd_90_/C) was reacted with the monometallic Au_10_/C catalyst, which we attribute to the poor ability of the Au_10_/C catalyst to catalyze the ORR. Strikingly, a significant CORE effect was observed when Au_90_–Pd_10_/C was reacted with the Pd_90_/C (HMF conversion increased from 67.0 to 91.3%) and the Pd_10_/C catalyst (HMF conversion increased from 26.3 to 58.7%).

It is evident that CORE is influenced by many different factors, some of which remain unclear. Based on the data presented herein, we can however state with confidence that CORE is influenced by (i) the rates of DH and ORR occurring on separate (coupled) sites which is governed by particle size effects and (ii) the ease with which electrons can be transferred from one component to the other. This supports our previous hypothesis, that electron transfer between components can proceed either directly or through the support (in Au@Pd/C type systems) or through physical contact between support grains in Au/C + Pd/C physical mixtures. The rate of the ORR and DH over Au or Pd sites in coupled systems, a factor that appears to be dependent on the support metal particle size, results in differences in the electrochemical potential of the two half-reactions. This hypothesis is further supported by the work of Tada and co-workers, who proposed that this electron transport between small and large Au nanoparticles in photocatalytic H_2_O_2_ synthesis proceeded in a similar manner.^46^

Importantly, it is still not understood whether CORE effects influence selective oxidation in such systems. Given that we strongly suspect that the structural properties of the catalysts in each series change as a function of metal weight loading, it is not possible to draw conclusions from the selectivity data presented in Tables S2–S6. For this reason, additional experiments were conducted over selected monometallic Au/C and Pd/C catalysts and the corresponding physical mixtures (Au_mol_/Pd_mol_ = 4, 1, and 0.25). Reaction times were carefully adjusted so that selectivity could be assessed at more comparable HMF conversion. The results from these experiments (Table S14) demonstrate that under Au-rich and equimolar conditions, CORE effects do appear to influence reaction selectivity. In both situations, HMFCA selectivity is reduced when both monometallic catalysts (Au/C and Pd/C) are combined in the same reactions. As a consequence, the selectivity to FFCA and FDCA in these reactions increases. No such correlation was observed with the series of catalysts that were Pd-rich (Au_mol_/Pd_mol_ = 0.25). Based on our hypothesis, that the Au and Pd roles are interchangeable (dependent on the Au/Pd ratio and supported metal particle size), it could imply that CORE directly influences reaction selectivity and is dependent on the specific properties of the metal that catalyzed the DH half-reaction. These are, of course, preliminary assumptions and a full independent study are required to validate this hypothesis.

The observation that the optimum alloy composition (Au_50_–Pd_50_/C) is more active than the optimum phase-separated catalyst (Au_67_@Pd_33_/C) and physical mixture (Au_50_/C + Pd_50_/C), does not diminish the impact of our findings. The discovery that the DH of alcohols and formyls can be considered as two separated, but complementary, catalytic processes will alter researchers’ approach to catalyst design. Understanding which of these processes is limiting provides a higher level of control and ultimately allows for significant improvements in catalytic performance to be tuned.

## Conclusions

This work demonstrates that CORE effects in coupled DH systems exist across a broad range of Au and Pd ratios. It also highlights that to predict the extent of a CORE effect, careful mechanistic considerations of potential coupling reactions are required. Here, we hypothesize that the DH of HMF (and reaction intermediates) can couple two and four-electron reduction processes and H_2_O_2_ reduction. The reaction(s) that couple with DH appears to be dependent on several features, including the relative amounts of Au and Pd present and the proximity of Au and Pd in the system under some Au/Pd ratios. Importantly, we infer that the magnitude of CORE effects and the roles of the two coupled metals are influenced by structural properties, such as the supported metal particle sizes. Despite the progress made, controlling, and understanding CORE effects in bimetallic catalysis is evidently challenging. We acknowledge that further work is required to fully understand the detailed mechanistic features of these enhancements, which remain largely unknown. The ability to harness these effects is an exciting prospect indeed and has the potential to influence both academic and industrial methodologies.

## Supporting Information Available

CV results; TEM images of Au/C and Pd/C catalysts; XPS data of Au–Pd/C and Au@Pd/C catalysts; time on line tests; specific activity data of each catalyst; and onset potentials from CV and ORR curves (PDF)
