# Supplementary material for: Insights into the Effect of Metal Ratio on Cooperative Redox Enhancement Effects over Au- and Pd-Mediated Alcohol Oxidation
Source: ACS Catal. 2023 Feb 10;13(5):2892–903. doi: 10.1021/acscatal.2c06284 (PMC9990151; doi:10.1021/acscatal.2c06284)
Supplement: Supplementary file 1 — cs2c06284_si_001.pdf [file cs2c06284_si_001.pdf]

Insights into the effect of metal ratio on co-operative redox enhancement effects over Au and Pd mediated alcohol oxidation

‡Liang Zhao[1], ‡Ouardia Akdim[1], ‡Xiaoyang Huang[1], Kai Wang[1], Mark Douthwaite[1]*, Samuel Pattisson[1], Richard J. Lewis[1], Runjia Lin[1], Bingqing Yao[2], David J. Morgan[1], Greg Shaw,[1] Qian He[2], Donald Bethell[1], Steven McIntosh[3], Christopher J. Kiely[3,4] and Graham J. Hutchings[1]**

‡These authors contributed equally

[1] Max Planck- Cardiff Centre on the Fundamentals of Heterogeneous Catalysis FUNCAT, Cardiff Catalysis Institute, School of Chemistry, Cardiff University, Cardiff, CF10 3AT, UK.

[2] Department of Materials Science and Engineering, Faculty of Engineering, National University of Singapore, 119077, Singapore.

[3] Department of Chemical and Biomolecular Engineering, Lehigh University, Bethlehem, PA 18015, USA.

[4] Department of Materials Science and Engineering, Lehigh University, Bethlehem, PA 18015, USA.

Corresponding authors E-mail: akdimo@cardiff.ac.uk, douthwaitejm@cardiff.ac.uk and hutch@cardiff.ac.uk

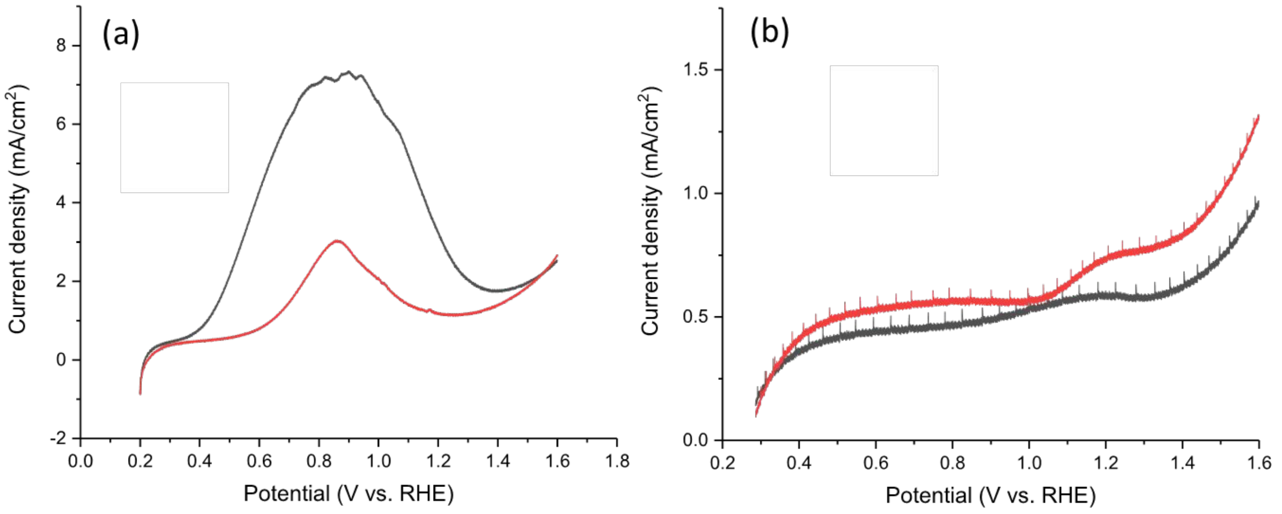

Figure S1. Cyclic voltammetry (anodic scan) of half-cell with HMF solution for catalyst series in the presence of (a) 0.1 M NaOH (*ca.* pH 14) and (b) 0.002 M NaOH (*ca.* pH 9). Reaction conditions: [NaOH] stated; 0.02 M HMF; 50 ml H_2O; 25 °C; scan rate, 50 mV . s^{-1}; N_2 flow, 50 mL . min^{-1}. **KEY**: Au_{50}/C (red line); Pd_{50}/C (black line).

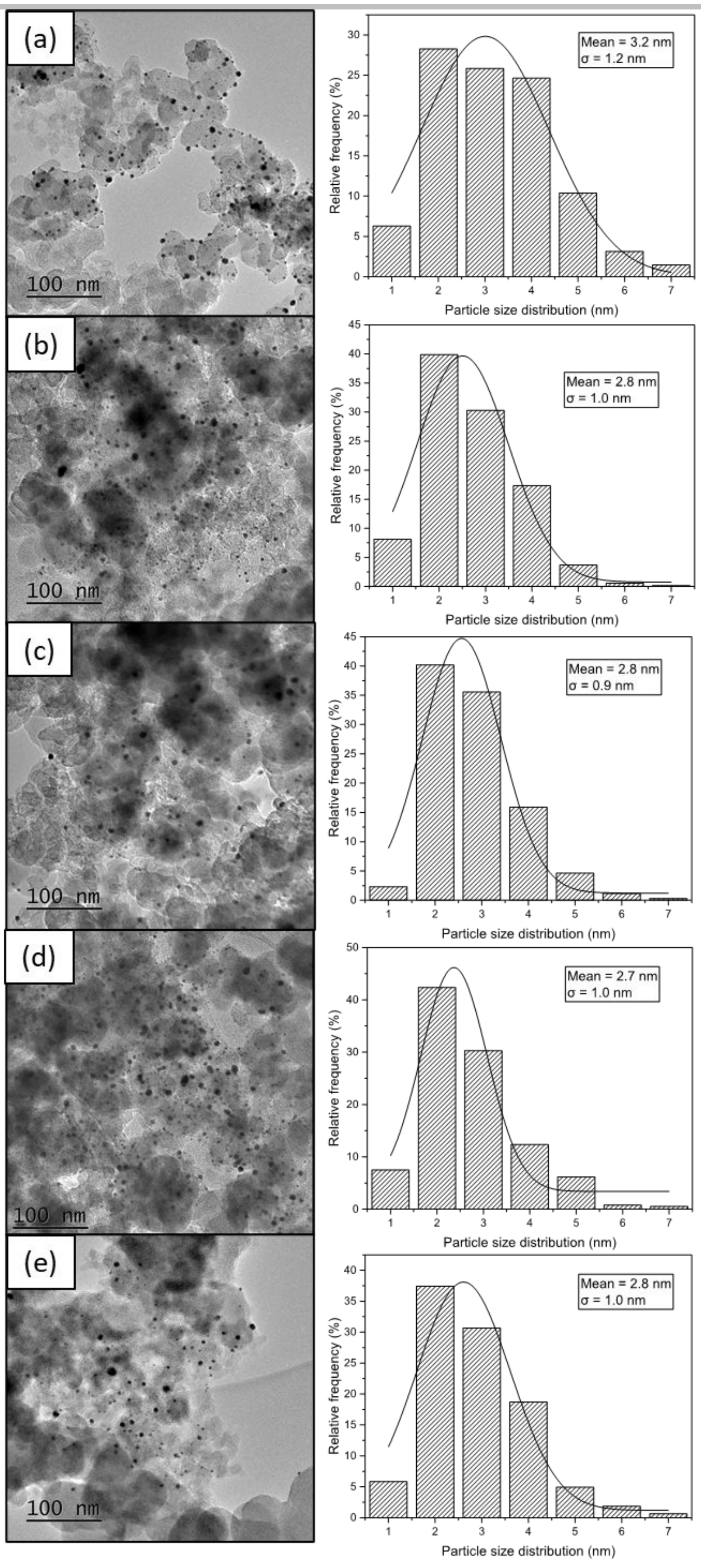

Figure S2. TEM micrographs and particle histograms for variety of Au/C supported catalysts: **KEY**: (a) Au_{80}/C; (b) Au_{67}/C; (c) Au_{50}/C; (d) Au_{33}/C; (e) Au_{20}/C.

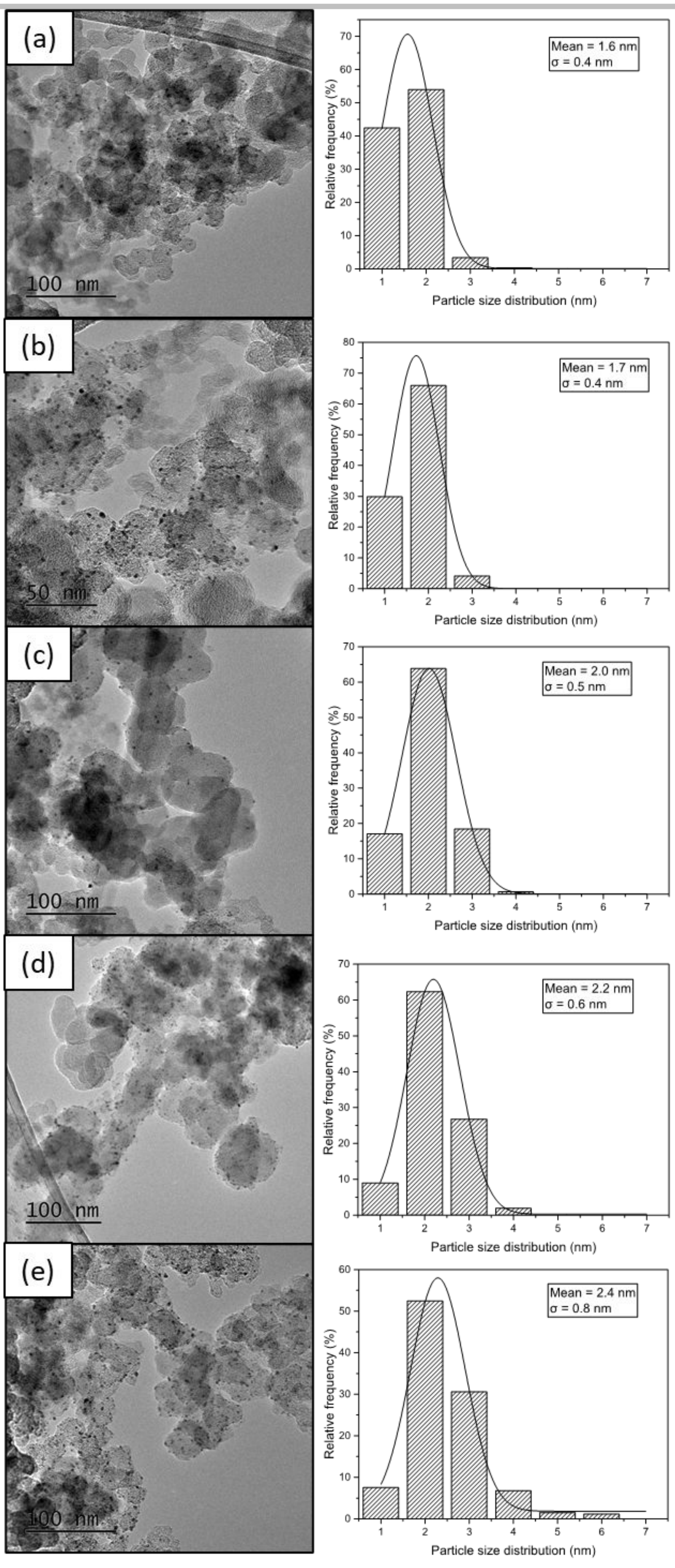
(a)
100 nm
Mean = 1.6 nm
σ = 0.4 nm
Relative frequency (%)
Particle size distribution (nm)
(b)
50 nm
Mean = 1.7 nm
σ = 0.4 nm
Relative frequency (%)
Particle size distribution (nm)
(c)
100 nm
Mean = 2.0 nm
σ = 0.5 nm
Relative frequency (%)
Particle size distribution (nm)
(d)
100 nm
Mean = 2.2 nm
σ = 0.6 nm
Relative frequency (%)
Particle size distribution (nm)
(e)
100 nm
Mean = 2.4 nm
σ = 0.8 nm
Relative frequency (%)
Particle size distribution (nm)

Figure S3. TEM micrographs and particle histograms for variety of Au/C supported catalysts: **KEY**: (a) Pd_{20}/C; (b) Pd_{33}/C; (c) Pd_{50}/C; (d) Pd_{67}/C; (e) Pd_{80}/C.

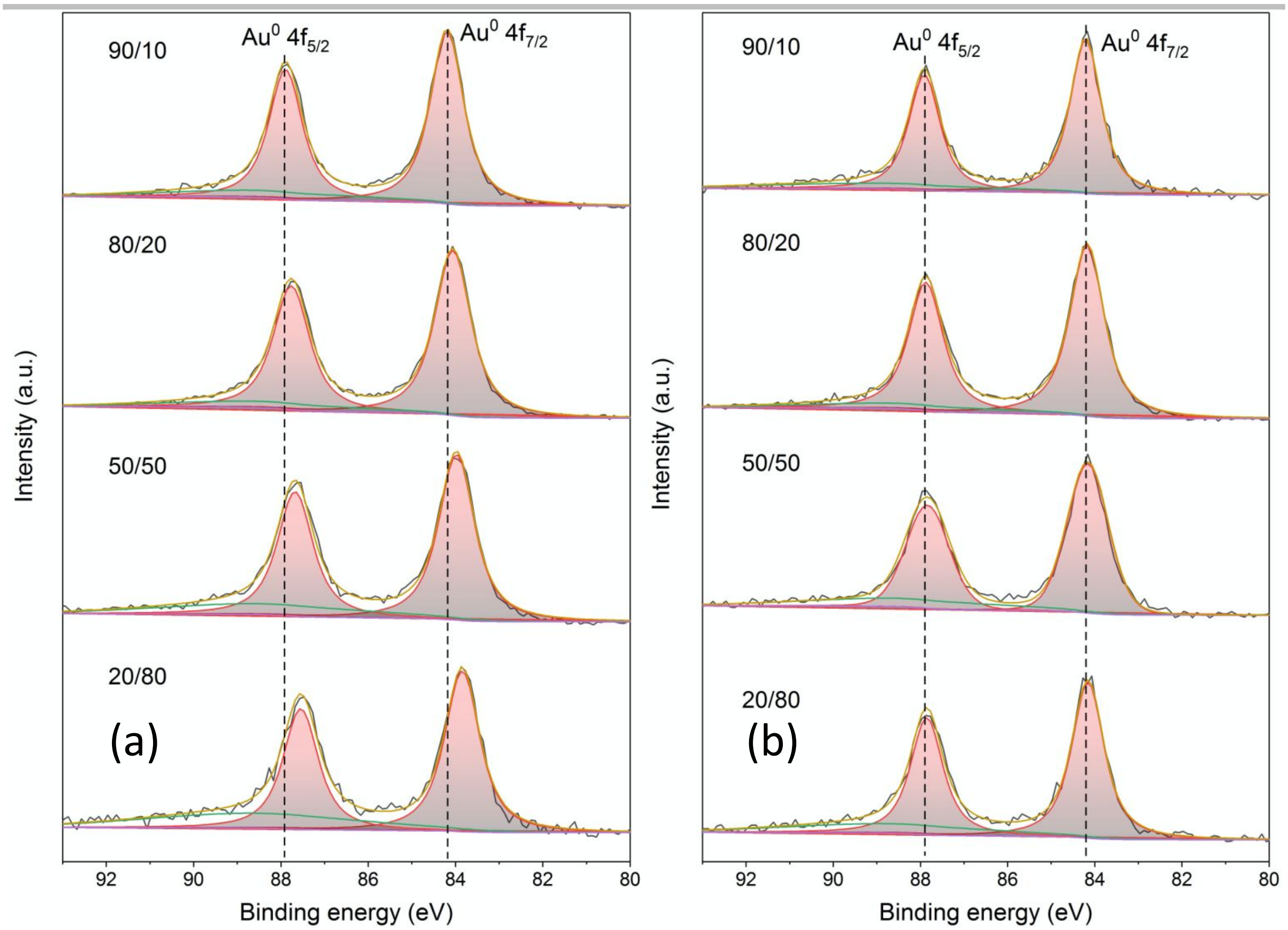

Figure S4. Au*4f* spectra from XPS experiments conducted over a series of (a) Au-Pd/C alloy catalysts and (b) Au@Pd/C catalysts with varying Au and Pd ratios.

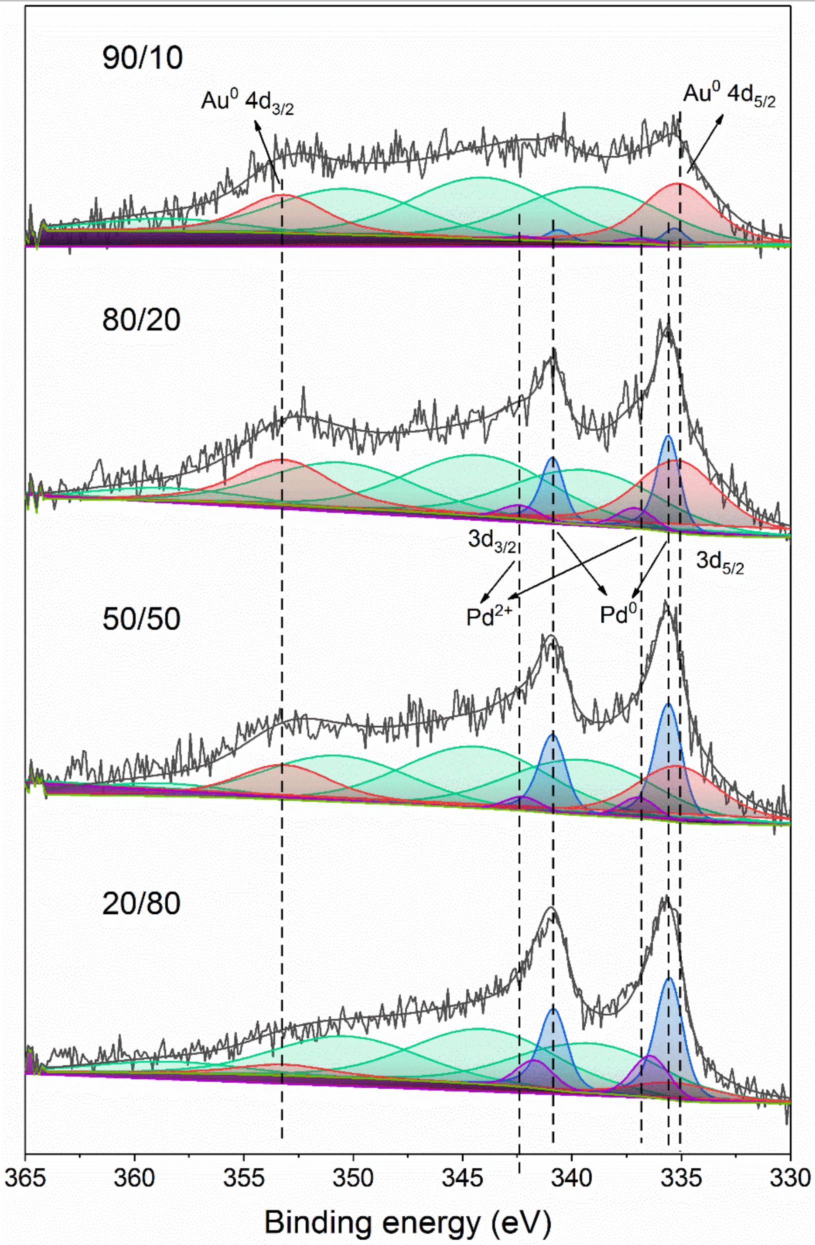

Figure S5. Pd*3d* spectra from XPS experiments conducted over a series of Au-Pd/C alloy catalysts with varying Au and Pd ratios, revealing Au^0*4d* (red), Pd^0*3d* (blue), Pd^{2+}*3d* (purple) and carbon loss (green) component, respectively.

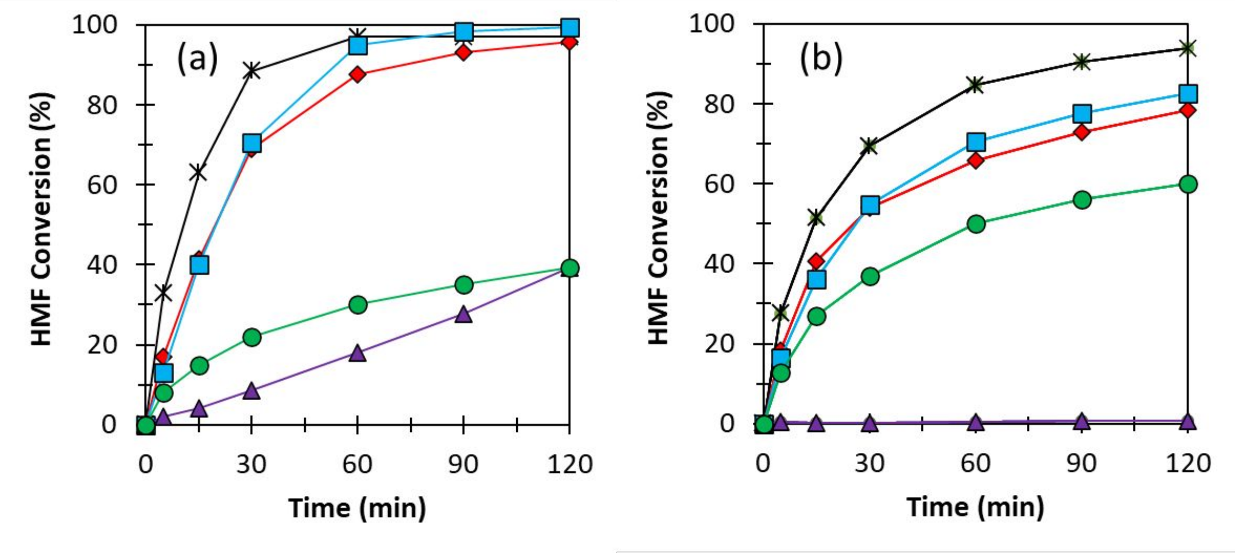

Figure S6. HMF conversion as a function of time over for catalyst series at the (a) equimolar (Au_{50}/Pd_{50}) and (b) Pd-rich (Au_{20}/Pd_{80}) regions. Reaction conditions: 0.1 M HMF; 0.4 M $NaHCO_3$ 16 mL H_2O; 80 °C; pO_2 =3 bar; time stated. **KEY**: Au/C (purple line/triangles); Pd/C (green line/circles); Au/C + Pd/C (blue line/squares); Au@Pd/C (red line/diamonds); Au-Pd/C (black line/crosses).

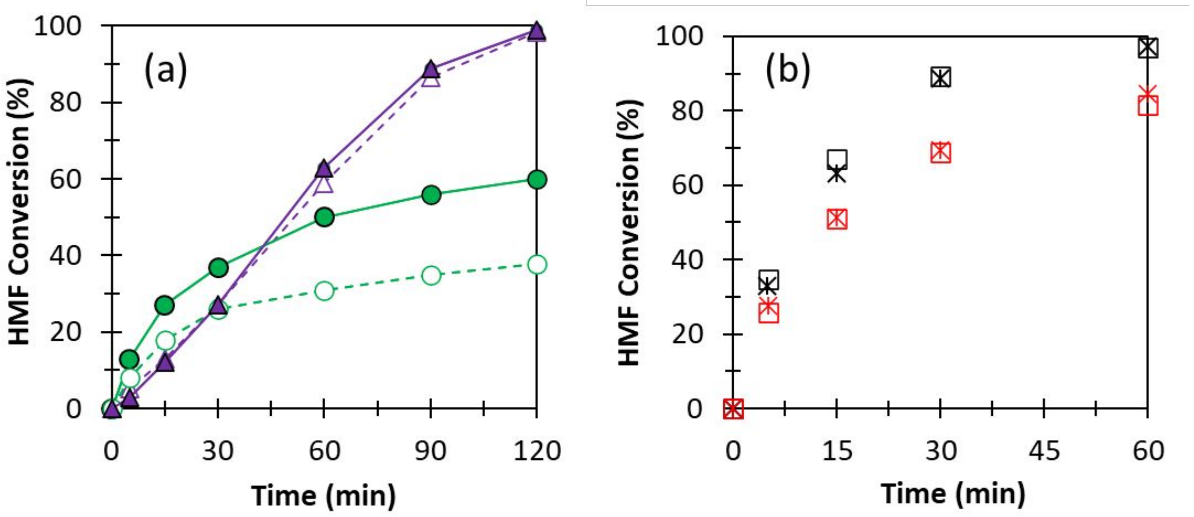

Figure S7. HMF conversion as a function of time over for different catalyst series in the presence and absence of FFCA (0.01 M). (a) HMF conversion over time of Pd_{80}/C in the presence (hollow green circles) and absence (filled green circles) of FFCA is compared with HMF conversion over time with Au_{80}/C in the presence (hollow purple triangles) and absence (filled purple triangles) of FFCA. (b) HMF conversion over time of Au_{50}-Pd_{50}/C in the presence (hollow black squares) and absence (black crosses) of FFCA is compared with HMF conversion over time with Au_{20}-Pd_{80}/C in the presence (hollow red squares) and absence (red crosses) of FFCA. Reaction conditions: 0.1 M HMF; 0.4 M $NaHCO_3$ 16 mL H_2O; 80 °C; pO_2 =3 bar; time stated min.

Table S1. Nomenclature for different catalysts used in this study and their theoretic Au and/or Pd weight loadings are provided. In cases where catalysts were analysed by TEM, mean particle sizes and the figures associated standard deviation is provided.

Catalyst	Catalyst Type	Metal Loading (wt.%)		Mean Particle Size (nm)	Particle Size Deviation (σ)
		Gold	Palladium		
Au_{100}/C	Mono	1.98		n.d.	n.d.
Au_{90}/C	Mono	1.85		n.d.	n.d.
Au_{80}/C	Mono	1.75		3.2	1.2
Au_{67}/C	Mono	1.55		2.8	1
Au_{50}/C	Mono	1.28		2.7	1
Au_{33}/C	Mono	0.95		2.8	0.9
Au_{20}/C	Mono	0.62		2.8	1
Au_{10}/C	Mono	0.34		n.d.	n.d.
Pd_{100}/C	Mono		1.98	n.d.	n.d.
Pd_{90}/C	Mono		1.63	n.d.	n.d.
Pd_{80}/C	Mono		1.35	2.4	0.8
Pd_{67}/C	Mono		1.03	2.2	0.6
Pd_{50}/C	Mono		0.7	2	0.5
Pd_{33}/C	Mono		0.42	1.7	0.4
Pd_{20}/C	Mono		0.24	1.6	0.4
Pd_{10}/C	Mono		0.11	n.d.	n.d.
Au_{90}-Pd_{10}/C	Alloy	0.94	0.06	n.d.	n.d.
Au_{80}-Pd_{20}/C	Alloy	0.88	0.12	2.8[a]	1.3[a]
Au_{67}-Pd_{33}/C	Alloy	0.78	0.22	n.d.	n.d.
Au_{50}-Pd_{50}/C	Alloy	0.64	0.36	n.d.	n.d.
Au_{33}-Pd_{67}/C	Alloy	0.47	0.53	n.d.	n.d.
Au_{20}-Pd_{80}/C	Alloy	0.31	0.69	n.d.	n.d.
Au_{10}-Pd_{90}/C	Alloy	0.17	0.83	n.d.	n.d.
Au_{90}@Pd_{10}/C	BM	0.94	0.06	n.d.	n.d.
Au_{80}@Pd_{20}/C	BM	0.88	0.12	4.5[a]	1.4[a]
Au_{67}@Pd_{33}/C	BM	0.78	0.22	2.2	0.8
Au_{50}@Pd_{50}/C	BM	0.64	0.36	2.7	1.1
Au_{33}@Pd_{67}/C	BM	0.47	0.53	n.d.	n.d.
Au_{20}@Pd_{80}/C	BM	0.31	0.69	2.1	0.9
Au_{10}@Pd_{90}/C	BM	0.17	0.83	n.d.	n.d.

Note: All catalysts prepared by variation of the same sol-immobilisation method. Particle size measurements were determined by electron microscopy (minimum 300 particle sample size). ***KEY*:** Mono (*supported monometallic particles*); Alloy (*supported alloyed particles*); BM (*spatially separated binary mixtures of supported Au and Pd*); C (*carbon XC-72R support*); [a] From previous paper;[1] n.d. stands for not determined.

Table S2. Activity and yield data for the aerobic oxidation of HMF over various carbon supported Pd catalysts, with different Pd weight loadings.

**Catalyst ID**	**HMF Conversion (%)**	**Activity$_{STO}$ (x 10^{-7} mol . s^{-1})**	**Yield (%)**			**CMB (%)**
			HMFCA	FFCA	FDCA	
Pd_{100}/C	50.3	7.4	20.9	21.5	2.6	96.0
Pd_{90}/C	41.9	6.2	19.5	18.8	2.2	97.0
Pd_{80}/C	32.6	5.0	18.7	14.7	1.7	98.0
Pd_{67}/C	22.0	3.0	14.3	7.1	0.5	98.0
Pd_{50}/C	21.7	2.8	13.3	6.8	0.5	98.0
Pd_{33}/C	15.8	2.1	9.6	3.5	0.2	98.0
Pd_{20}/C	5.9	0.3	3.2	0.8	0.0	97.0
Pd_{10}/C	1.0	< 0.1	0.1	0.0	0.0	99.0

Reaction Conditions: 0.1 M HMF; 0.4 M $NaHCO_3$ 16 mL H_2O; 80 °C; pO_2 =3 bar; 30 min. **KEY:** 5-Hydroxymethyl-2-furancarboxylic acid (HMFCA); 5-Formyl-2-furancarboxylic acid (FFCA); 2,5-Furan dicarboxylic acid (FDCA); Carbon mass balance (CMB).

Table S3. Activity and yield data for the aerobic oxidation of HMF over various carbon-supported Au catalysts, with different Au weight loadings.

Catalyst ID	Conversion (%)	Activity$_{STO}$ (x 10^{-7} mol . s^{-1})	Yield (%)			CMB (%)
			HMFCA	FFCA	FDCA	
Au_{100}/C	23.4	2.1	13.7	4.5	0.1	96
Au_{90}/C	18.2	1.5	11.3	2.5	0.1	95
Au_{80}/C	16.5	1.4	9.2	2.2	0.1	95
Au_{67}/C	9.5	1.0	6.4	1.8	0.1	99
Au_{50}/C	6.9	0.78	6	1.1	0.1	98
Au_{33}/C	1.8	0.013	0.8	0.1	0	99
Au_{20}/C	0.8	0.002	0.1	0	0	100
Au_{10}/C	0	0	0	0	0	100

Reaction Conditions: 0.1 M HMF; 0.4 M $NaHCO_3$ 16 mL H_2O; 80 °C; pO_2 =3 bar; 30 min. **KEY:** 5-5-Hydroxymethyl-2-furancarboxylic acid (HMFCA); 5-Formyl-2-furancarboxylic acid (FFCA); 2,5-Furan dicarboxylic acid (FDCA); Carbon mass balance (CMB).

Table S4. Activity and yield data for the aerobic oxidation of HMF over various physical mixtures of monometallic carbon-supported Au and Pd catalysts.

Catalyst ID	HMF Conversion (%)	Activity$_{STO}$ (x 10^{-7} mol . s^{-1})	Yield (%)			CMB (%)
			HMFCA	FFCA	FDCA	
$Au_{90}/C + Pd_{10}/C$	16.8	1.4	7.7	3.2	0.1	95.0
$Au_{80}/C + Pd_{20}/C$	50.2	5.6	27.1	15.8	0.8	95.0
$Au_{67}/C + Pd_{33}/C$	59.2	8.3	29.3	26.2	1.6	97.0
$Au_{50}/C + Pd_{50}/C$	69.7	9.9	32.8	29.8	3.3	96.0
$Au_{33}/C + Pd_{67}/C$	63.9	9.7	29.4	28.6	3.4	97.0
$Au_{20}/C + Pd_{80}/C$	50.7	6.9	23.7	23.0	3.3	95.0
$Au_{10}/C + Pd_{90}/C$	50.5	6.7	23.2	22.5	2.8	97.0

Reaction Conditions: 0.1 M HMF; 0.4 M $NaHCO_3$ 16 mL H_2O; 80 °C; pO_2 =3 bar; 30 min. **KEY:** 5-Hydroxymethyl-2-furancarboxylic acid (HMFCA); 5-Formyl-2-furancarboxylic acid (FFCA); 2,5-Furan dicarboxylic acid (FDCA); Carbon mass balance (CMB).

Table S5. Activity and yield data for the aerobic oxidation of HMF over various carbon-supported Au@Pd/C alloy catalysts, with different Au/Pd weight loadings.

**Catalyst ID**	**HMF Conversion (%)**	**Activity$_{STO}$ (x 10^{-7} mol . s^{-1})**	**Yield (%)**			**CMB (%)**
			**HMFCA**	**FFCA**	**FDCA**	
Au_{90}@Pd_{10}/C	6.4	0.3	1.4	0.7	0.0	96.0
Au_{80}@Pd_{20}/C	65.5	8.7	36.1	26.5	1.8	97.0
Au_{67}@Pd_{33}/C	79.9	12.6	24.8	50.1	4.5	97.0
Au_{50}@Pd_{50}/C	69.5	10.9	17.5	45.1	3.3	96.0
Au_{33}@Pd_{67}/C	59.9	8.5	15.1	34.2	3.2	95.0
Au_{20}@Pd_{80}/C	52.4	7.7	14.5	32.5	3.2	95.0
Au_{10}@Pd_{90}/C	51.3	6.9	13.2	27.2	2.2	93.0

Reaction Conditions: 0.1 M HMF; 0.4 M $NaHCO_3$ 16 mL H_2O; 80 °C; pO_2 =3 bar; 30 min. **KEY:** 5-Hydroxymethyl-2-furancarboxylic acid (HMFCA); 5-Formyl-2-furancarboxylic acid (FFCA); 2,5-Furan dicarboxylic acid (FDCA); Carbon mass balance (CMB).

Table S6. Activity and yield data for the aerobic oxidation of HMF over various carbon-supported Au-Pd/C alloy catalysts, with different Au/Pd weight loadings.

Catalyst ID	Conversion (%)	$Activity_{STO}$ (x 10^{-7} mol . s^{-1})	Yield (%)			CMB (%)
			HMFCA	FFCA	FDCA	
Au_{90}-Pd_{10}/C	21.1	2.0	12.8	4.4	0.1	97.0
Au_{80}-Pd_{20}/C	42.3	5.0	26.3	14.4	0.4	96.0
Au_{67}-Pd_{33}/C	79.8	10.4	31.2	37.7	2.2	95.0
Au_{50}-Pd_{50}/C	89.5	14.2	22.6	57.5	5.8	97.0
Au_{33}-Pd_{67}/C	78.4	12.3	19.1	49.6	5.1	96.0
Au_{20}-Pd_{80}/C	69.6	10.7	15.6	44.2	4.4	95.0
Au_{10}-Pd_{90}/C	63.4	9.5	15.5	37.9	3.8	96.0

Reaction Conditions: 0.1 M HMF; 0.4 M $NaHCO_3$ 16 mL H_2O; 80 °C; pO_2 =3 bar; 30 min. **KEY:** 5-Hydroxymethyl-2-furancarboxylic acid (HMFCA); 5-Formyl-2-furancarboxylic acid (FFCA); 2,5-Furan dicarboxylic acid (FDCA); Carbon mass balance (CMB).

Table S7. pH over the time course of the HMF oxidation across the 50/50 Au/Pd ratio.

Catalyst ID	pH online test						
	0 min	5 min	15 min	30 min	60 min	90 min	120 min
Carbon	8.9	9.2	9.3	9.3	9.3	9.3	9.3
Au_{50}-Pd_{50}/C	8.9	8.8	8.8	8.7	8.7	8.8	8.9
Au_{50}@Pd_{50}/C	8.9	9.1	8.9	8.8	8.8	8.9	8.9
Au_{50}/C+Pd_{50}/C	8.9	9.2	9.0	8.9	8.8	8.9	8.9
Au_{50}/C	8.9	9.2	9.3	9.4	9.4	9.4	9.3
Pd_{50}/C	8.9	9.2	9.3	9.2	9.2	9.2	9.1

Reaction conditions: 0.1 M HMF; 0.4 M $NaHCO_3$; 16 mL H_2O; 80 °C; pO_2 =3 bar; 120 min

Table S8. Quantification of H_2O_2 generated in-situ during the aqueous phase of HMF oxidation.

Catalyst	H_2O_2 detected (ppm)
Au_{80}/C	28±1[a]
Au_{20}/C	0
Pd_{80}/C	0
Pd_{20}/C	0
Au_{80}/C + Pd_{20}/C	0
Au_{80}/C + Pd_{20}/C	0

Reaction conditions: 0.1 M HMF; 0.4 M $NaHCO_3$; 16 mL H_2O; 80 °C; pO_2 =3 bar; 15 min.

[a.] Standard error was obtained by repeating three times.

Table S9. Comparison of CORE effects exhibited at low conversion and after 30 minutes of reaction.

Catalysts	Conversion (%)	Reaction Time (min)	Rate (mol/L/min)	CORE Mag at initial rates	CORE Mag at 30 min
Au_{80}/C	12.2	15	8.1×10^{-4}		
Pd_{20}/C	6.1	30	2.0×10^{-4}		
$Au_{80}/C+Pd_{20}/C$	4.5	2	2.3×10^{-3}	2.21	2.25
Au_{50}/C	8.7	30	2.9×10^{-4}		
Pd_{50}/C	8.2	5	1.6×10^{-3}		
$Au_{50}/C+Pd_{50}/C$	8.2	2	4.1×10^{-3}	2.12	2.43
Au_{20}/C	0.2	30	7.6×10^{-6}		
Pd_{80}/C	7.0	2	3.5×10^{-3}		
$Au_{20}/C+Pd_{80}/C$	9.9	2	5.0×10^{-3}	1.41	1.54

CORE Magnitude = (reaction rate of physical mixture of Au/C and Pd/C) / (rate sum of Au/C and Pd/C)

Reaction conditions: 0.1 M HMF; 0.4 M $NaHCO_3$; 16 mL H_2O; 80 °C; pO_2 =3 bar; Time = stated

Table S10. Onset potentials acquired from CV scans over various catalysts in the presence of HMF

Catalyst	Onset Potential (V)
Au_{50}/C	0.38
Pd_{50}/C	0.53
$Au_{50}/C + Pd_{50}/C$	0.51
$Au_{50}@Pd_{50}/C$	0.51
Au_{50}-Pd_{50}/C	0.35
Au_{20}/C	0.42
Pd_{80}/C	0.47
$Au_{20}/C + Pd_{80}/C$	0.47
$Au_{20}@Pd_{80}/C$	0.47
Au_{20}-Pd_{80}/C	0.47

Reaction conditions: 0.1 M NaOH; 0.02 M HMF; 50 ml H_2O; 25 °C; scan rate, 50 mV . s^{-1}; O_2 flow, 50 mL . min^{-1}.

Table S11. Onset potentials acquired from ORR polarisation curves for monometallic Au/C and Pd/C catalysts with and without HMF.

Catalyst	Onset Potential (V)	
	No HMF	HMF
Pd_{50}/C	0.81 ± 0.02	0.86 ± 0.01
Pd_{80}/C	0.79 ± 0.01	0.87 ± 0.01
Au_{50}/C	0.73 ± 0.01	0.74 ± 0.00
Au_{20}/C	0.74 ± 0.01	0.74 ± 0.00

Reaction Conditions: 0.1 M NaOH; 0.02 M HMF (or without); 50 ml H_2O; 25 °C; O_2 flow, 50 mL.min^{-1}.

Error margins acquired from running a minimum of two experiments; the value presented is indicative of the mean onet-potential acquired from these experiments.

Table S12. Thermocatalytic HMF oxidation activity under electrochemical reaction conditions.

Catalyst	Conv. (%) at 5 mins	Conv. (%) At 15 mins	CORE Enhancement (%)	CMB (%)
Blank	0	0		99
Au_{80}/C	32	-		96
Pd_{20}/C	2.6	-		96
$Au_{80}/C+Pd_{20}/C$	54.1	-	19.5	95
Au_{50}/C	18.9	-		95
Pd_{50}/C	4.1	-		96
$Au_{50}/C+Pd_{50}/C$	28.4	-	5.4	95
Au_{20}/C	-	7.4		96
Pd_{80}/C	-	16.8		95
$Au_{20}/C+ Pd_{80}/C$		28.2	4.0	95

Reaction conditions: 0.1 M NaOH, 0.02 M HMF, 35 mL H_2O, O_2 50 mL . min^{-1}, room temperature.
KEY: Carbon mass balance (CMB).

CORE enhancement = (Conversion of physical mixture) - (conversion sum of Au/C + Pd/C).

Table S13. $Activity_{STO}$ of the monometallic Au_{20}/C and Au_{80}/C catalysts for thermocatalytic HMF oxidation is presented.

Catalyst ID	Catalyst Mass (mg)	$Activity_{STO}$ (x 10^{-7} $mol.s^{-1}$)
Au_{20}/C	71	0.008
Au_{20}/C	140	0.01
Au_{80}/C	72	1.4
Au_{80}/C	144	3.2

Reaction conditions: 0.1 M HMF; 0.4 M $NaHCO_3$; 16 mL H_2O; 80 °C; pO_2 =3 bar; time = 30 mins.

Table S14. Reaction selectivity is monitored as a function over conversion over a series of monometallic catalysts and analogous physical mixtures.

Catalyst	Reaction Time (min)	Conversion (%)	Selectivity (%)		
			HMFCA	FFCA	FDCA
Au_{80}/C	30	16.9	80.2	19.2	0.6
Pd_{20}/C	30	6.1	79.8	20.2	0.0
$Au_{80}/C + Pd_{20}/C$	15	24.2	62.0	36.1	1.9
Au_{50}/C	120	39.4	83.1	15.7	1.2
Pd_{50}/C	120	39.2	66.4	30.1	3.5
$Au_{50}/C + Pd_{50}/C$	15	40.0	54.1	40.6	5.3
Au_{20}/C	120	0.8	100.0	0.0	0.0
Pd_{80}/C	30	36.9	53.2	41.8	5.0
$Au_{20}/C+Pd_{80}/C$	15	36.1	52.3	42.6	5.1

Reaction conditions: 0.1 M HMF; 0.4 M $NaHCO_3$; 16 mL H_2O; 80 °C; pO_2 =3 bar; time = stated.

References

(1) Huang, X.; Akdim, O.; Douthwaite, M.; Wang, K.; Zhao, L.; Lewis, R. J.; Pattisson, S.; Daniel, I. T.; Miedziak, P. J.; Shaw, G.; Morgan, D. J.; Althahban, S. M.; Davies, T. E.; He, Q.; Wang, F.; Fu, J.; Bethell, D.; McIntosh, S.; Kiely, C. J.; Hutchings, G. J. Au-Pd separation enhances bimetallic catalysis of alcohol oxidation. *Nature* **2022**, *603*, 271-275.
